# Financial risk protection from out-of-pocket health spending in low- and middle-income countries: a scoping review of the literature

**DOI:** 10.1186/s12961-022-00886-3

**Published:** 2022-07-29

**Authors:** Taslima Rahman, Dominic Gasbarro, Khurshid Alam

**Affiliations:** 1 Murdoch Business School, Murdoch University, Perth, WA 6150 Australia; 2 Institute of Health Economics, University of Dhaka, Dhaka, 1000 Bangladesh

**Keywords:** Out-of-pocket expenditure, Financial risk protection, Catastrophic health expenditure, Impoverishment, Coping strategies, Forgone care, Low- and middle-income countries

## Abstract

**Background:**

Financial risk protection (FRP), defined as households’ access to needed healthcare services without experiencing undue financial hardship, is a critical health systems target, particularly in low- and middle-income countries (LMICs). Given the remarkable growth in FRP literature in recent times, we conducted a scoping review of the literature on FRP from out-of-pocket (OOP) health spending in LMICs. The objective was to review current knowledge, identify evidence gaps and propose future research directions.

**Methods:**

We followed the Preferred Reporting Items for Systematic Reviews and Meta-Analyses (PRISMA) 2020 guidelines to conduct this scoping review. We systematically searched PubMed, Scopus, ProQuest and Web of Science in July 2021 for literature published since 1 January 2015. We included empirical studies that used nationally representative data from household surveys to measure the incidence of at least one of the following indicators: catastrophic health expenditure (CHE), impoverishment, adoption of strategies to cope with OOP expenses, and forgone care for financial reasons. Our review covered 155 studies and analysed the geographical focus, data sources, methods and analytical rigour of the studies. We also examined the level of FRP by disease categories (all diseases, chronic illnesses, communicable diseases) and the effect of health insurance on FRP.

**Results:**

The extant literature primarily focused on India and China as research settings. Notably, no FRP study was available on chronic illness in any low-income country (LIC) or on communicable diseases in an upper-middle-income country (UMIC). Only one study comprehensively measured FRP by examining all four indicators. Most studies assessed (lack of) FRP as CHE incidence alone (37.4%) or as CHE and impoverishment incidence (39.4%). However, the LMIC literature did not incorporate the recent methodological advances to measure CHE and impoverishment that address the limitations of conventional methods. There were also gaps in utilizing available panel data to determine the length of the lack of FRP (e.g. duration of poverty caused by OOP expenses). The current estimates of FRP varied substantially among the LMICs, with some of the poorest countries in the world experiencing similar or even lower rates of CHE and impoverishment compared with the UMICs. Also, health insurance in LMICs did not consistently offer a higher degree of FRP.

**Conclusion:**

The literature to date is unable to provide a reliable representation of the actual level of protection enjoyed by the LMIC population because of the lack of comprehensive measurement of FRP indicators coupled with the use of dated methodologies. Future research in LMICs should address the shortcomings identified in this review.

**Supplementary Information:**

The online version contains supplementary material available at 10.1186/s12961-022-00886-3.

## Background

Financial risk protection (FRP), defined as the ability to consume needed quality healthcare services without experiencing undue financial hardship, is one of the critical components of universal health coverage (UHC), an agreed target of the United Nations (UN) Sustainable Development Goal (SDG) 3 on health and well-being [1, 2]. A health system that protects people from financial risks ensures that the consumption of needed healthcare will improve their health status without compromising their economic well-being [3]. In the absence of adequate FRP, people needing to pay out-of-pocket (OOP) at the point of service are at risk of undergoing a range of adverse health and economic consequences. The damaging upshots may include limited or no access to healthcare, deteriorated health status, the undesirable substitution of essential consumption in the current or future period due to depleted household assets, slipping into or spiralling deep into poverty, and intensified health and socioeconomic inequalities.

The literature captures the undesirable economic effects of the OOP mode of payment for healthcare through one or more of the following indicators: catastrophic health expenditure (CHE), impoverishment, adoption of coping strategies and forgone care for financial reasons (FCFR) [3–9]. The incidence of CHE is measured as the percentage of households whose OOP healthcare expenses surpass a predetermined proportion or threshold of their available resources to pay for healthcare, or capacity to pay (CTP) [3, 4]. The assumption here is that such an extent of OOP health expenses reduces household consumption of other nonmedical necessities (e.g. food) [3]. Impoverishment incidence is defined as the proportion of households that were above the poverty line (PL) (i.e. nonpoor) before paying for healthcare from OOP but found themselves below the PL (i.e. poor) after OOP expenses [3]. The incidence of the adoption of coping strategies or distress or hardship financing is measured by the percentage of households that borrow money, sell productive assets, draw from savings, seek contributions from friends and family, or any combination of the above to meet OOP expenses [6, 10, 11]. On the other hand, the incidence of FCFR measures the proportion of households that forgo healthcare because of high or unaffordable OOP costs of care [7, 12]. Notably, CHE is the sole indicator in the SDG framework (SDG 3.8.2) to track progress towards UHC [4].

CHE is measured in a variety of ways, with metrics differing in how household CTP is defined. Traditional methods of CHE measurement include the budget share method, actual food expenditure method and the normative food expenditure method. The budget share method equates CTP to the household’s entire budget (i.e. income or consumption expenditure) [13]. The latter two methods derive CTP by deducting an allowance for basic needs from the household budget. The actual food expenditure method subtracts a household’s actual food expenses from its budget and calculates CHE based on the residual amount (i.e. nonfood expenditure) [13]. The normative food expenditure method refines it by calculating a standard amount that households must spend on food, subtracting it from the total budget and calculating CHE based on the remainder (called standard food expenditure-based non-subsistence expenditure) [14]. However, the normative food expenditure method is no different from the actual food expenditure method when the household is poor; that is, the household’s budget is less than the standard food expenditure. In such a case, actual food expenses are deducted from the budget instead of the higher, standard spending. Empirical evidence shows that the three traditional methods cannot precisely identify poor households that experience financial hardship despite spending relatively small amounts on healthcare [15, 16]. Specifically, the budget share method is the least sensitive to financial hardship among poorer households, which tends to underestimate CHE among the poor and overestimate it among the wealthy [15, 16]. Therefore, the traditional methods of CHE measurement present a challenge to equity analysis and pro-poor policy initiatives [5, 16].

More refined measurement techniques were subsequently developed. Wagstaff and Eozenou’s method (2014), developed as part of a World Bank (WB) effort, calculates CTP by deducting the prevailing PL, thereby providing a link between CHE and poverty [17]. Depending on the PL employed, this is likely to yield a greater concentration of CHE among the poor than among the rich, compared with the budget share method [4]. The normative food, housing (rent) and utilities method developed (in 2016) by the WHO Regional Office for Europe deducts a relative PL representing standard expenditures on basic need items (food, housing [rent] and utilities) consistently from all households [4, 18]. Additionally, any OOP expense by poor households is considered both further impoverishing and catastrophic. The normative food, housing (rent) and utilities method is the only approach in which wealthier households are consistently required to spend a higher proportion of their budget on healthcare to be counted as incurring CHE [15]. According to empirical research, the resulting CHE estimate is more sensitive to financial hardship experienced by poor households than the other methods, producing actionable evidence for policy [15, 16].

Different CHE metrics employ different thresholds: the budget share method typically uses 10% or 25%, while the other four employ 25% and 40% thresholds [13, 14, 17, 18]. As the official indicator for SDG 3.8.2, CHE is based on the budget share method, with 10% and 25% thresholds [5]. Impoverishment metrics also vary in the PLs they use. Absolute PLs may include the WB’s international PL (currently $1.90 per capita per day in purchasing power parity) or national PLs based on the WB’s poverty assessment, food poverty (cost of minimum food requirements), or basic needs (cost of a basket of goods thought to meet minimum biological needs) [19]. Relative PLs may be based on income (a specific percentage of the country’s median income, e.g. 50%, 60%) or household spending on basic needs [19]. Notably, already poor households whose poverty was exacerbated by OOP health payments are not included in the measurement of impoverishment incidence. Some recent studies consider OOP expenses incurred by poor households as further impoverishing, and measure them as a separate indicator of the impoverishing effects of OOP expenses [17, 18, 20].

The issue of FRP has attracted great interest from the scientific community and has given rise to remarkable growth in the literature in recent times [21]. However, the growth of studies, particularly in the low- and middle-income countries (LMICs), has outpaced existing reviews, which are limited by time and scope. Among the previous reviews most germane to this study, Rijal et al. (2018) examined the financial burden of noncommunicable diseases (NCDs) in South Asia, focusing on OOP expenditure, CHE, impoverishment and coping strategies adopted by patients with NCDs and their families; Erlangga et al. (2019) investigated the impact of government health insurance on health status, healthcare utilization and FRP in LMICs; and Alam and Mahal (2014) focused on the household-level economic impact of health shocks in LMICs in the pre-2014 literature [22–24]. However, considerable gaps remain. In particular, the post-2014 LMIC literature on FRP from OOP payments has simply not been covered in the existing reviews.

We, therefore, set out to conduct a scoping review to summarize recent evidence on FRP in healthcare in the LMICs based on the WB classification of countries by income as of 2021 (i.e. low-income [LIC], lower-middle-income [LwMIC] and upper-middle-income countries [UMIC] with per capita gross national income of US$ 12,695 or less) [25]. The overarching research question steering this review is: how financially protected are the households in the LMICs when they need healthcare? The specific objectives are to review the methodologies used in the recent literature to measure FRP, to determine the level of FRP enjoyed by the LMIC population, and to determine differences across country income groups (LICs, LwMICs and UMICs) for both of the above. This review adds to previous reviews by drawing together a larger number of recent studies on household-level FRP in healthcare in the LMICs of Africa, Asia, south-eastern Europe and Latin America, specifically between 2015 and 2021. Additionally, advances in the methodology for measuring FRP developed in recent times mean that this study adds considerably to the information base on FRP in accessing healthcare.

## Methods

**Fig. 1:**
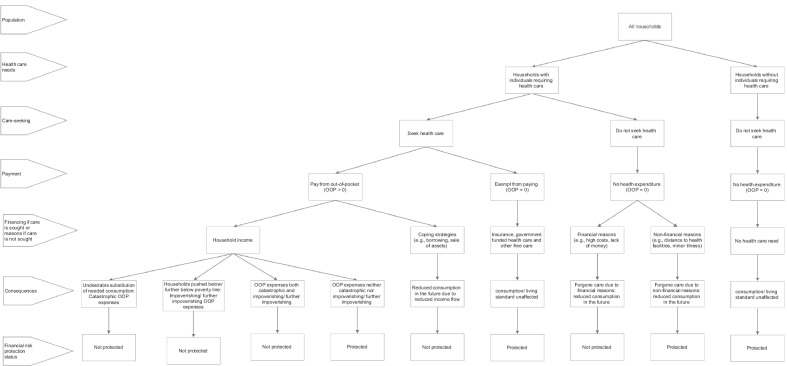
Conceptual framework of financial risk protection in LMICs

We developed a conceptual framework (Fig. 1) based on the FRP literature in healthcare to guide this review. We maintain that CHE, impoverishment effects, adoption of coping strategies and FCFR highlight distinct aspects of the lack of FRP in healthcare. Therefore, a comprehensive FRP study must measure all four indicators to determine how well a country is doing in ensuring FRP. Identifying the population lacking FRP through a smaller subset of these indicators is unlikely to fully reflect the intricacy of the notion of FRP, which could lead to inaccurate or misleading conclusions or, worse, create perverse incentives for policy-makers [26].

Figure 1 shows how a household’s FRP status can be measured by examining its healthcare needs, care-seeking behaviour, OOP payment requirement and financing source for OOP expenses if care is sought or reasons if not. To identify the households lacking FRP, we divide all households between those with and without healthcare needs (e.g. preventative, curative, rehabilitative, long-term, palliative). Households without healthcare needs do not pay anything towards healthcare and, therefore, do not lack FRP. We then partition families requiring care into those seeking and not seeking care. Households may fail to seek needed care for financial and nonfinancial reasons. We further separate the families seeking healthcare into those who pay and those who do not. Households may be exempt from paying if they are fully insured against healthcare costs or receive free care (e.g. from government, nongovernmental organizations, charities). Those who must pay OOP may finance it from their income or, if insufficient, adopt coping strategies (such as borrowing with or without interest or collateral, sale of assets).

Households that use their income to meet OOP expenses are not financially protected if the payments have catastrophic or impoverishing effects. Households incurring CHE lack FRP because OOP expenses relative to their available resources are so high that they forgo the consumption of other (welfare-augmenting) necessities [3]. On the other hand, households impoverished or further impoverished due to OOP expenses lack FRP because OOP expenses push them below or further below the PL, a threshold beneath which even the most basic standard of living is not guaranteed [3]. Thus, CHE and impoverishment are separate facets of a lack of FRP. Although some households may suffer both catastrophic and impoverishing effects of OOP expenses, global evidence suggests that this overlap is minimal (8–14%) [27]. Therefore, it is critical to complement CHE with impoverishment indicators to assess the degree of FRP enjoyed by a population.

Additionally, CHE and impoverishment indicators are blind to how households finance OOP expenses. Suppose families employ coping mechanisms such as taking out loans and selling assets to meet OOP expenses. In that case, they inflate household budgets and CTP, allowing them to pay for care while possibly preventing them from experiencing CHE and impoverishment in the short term. However, families employing coping techniques lack FRP, as these strategies might affect long-term household welfare by limiting the flow of income (because of loan repayments with interest or lost returns from the sale of productive assets) and the ability to cope again if needed [8].

Furthermore, CHE, impoverishment and coping metrics do not include households with zero OOP expenses; they show lack of FRP only among households that make OOP payments for healthcare. However, not all families with zero OOP expenses are financially protected. Households that forgo needed care (and thus have zero OOP expenditure) for financial reasons such as high or unaffordable cost of care seriously lack FRP. These might be the households with insufficient income that cannot seek healthcare even through coping. Forgoing care may exacerbate health problems and put the concerned families in a downward spiral of ill health and poverty [28, 29]. Hence, the extent to which OOP expenses prevent households from seeking necessary healthcare is another crucial indicator of FRP.

Therefore, we consider households as lacking FRP if they incur catastrophic or impoverishing OOP expenses, pay for healthcare through coping strategies or forgo healthcare altogether for financial reasons. We, however, recognize that catastrophic and impoverishing OOP hurt household economic welfare in the current period, and coping strategies and FCFR reduce welfare in the future. Failure to consider all four indicators to examine FRP may underestimate the overall economic impact on households of the requirement to make OOP payments for healthcare.

### Search strategies

We developed our protocol for this scoping review with support from Murdoch University (Australia) subject librarians (healthcare, and business and economics). Before the final data collection, we pilot-tested and calibrated the protocol to ensure its applicability. We searched for empirical literature on FRP in LMICs in the following electronic databases and platforms: PubMed, Scopus, ProQuest (EconLit and APA PsycInfo) and Web of Science (Social Sciences Citation Index). We used key search terms including “out-of-pocket expenditure, “financial risk protection”, “catastrophic health expenditure”, “impoverishment”, “coping” and “forgone care” along with the names of the countries in LIC, LwMIC and UMIC groups according to the WB country classification by per capita income in July 2021 [25]. The searches were carried out on 13 July 2021. Time, language and type of publication filters were used in the searches to identify articles published in peer-reviewed journals in the English language since 2015. The search strings are available in Additional file 1.

### Study selection

We followed the Preferred Reporting Items for Systematic Reviews and Meta-Analysis (PRISMA) 2020 guidelines in the study selection process [30]. The retrieved studies were uploaded to the bibliographic software EndNote 2020, which was used to identify duplicates by using the title, year and reference type, ignoring spacing and punctuation. Non-duplicate records were uploaded to the Rayyan web app for systematic reviews [31] to screen the titles and abstracts for assessing eligibility based on strict inclusion and exclusion criteria. The eligibility criteria were as follows: original research articles in scholarly journals; studies on countries in the WB’s list of LICs, LwMICs and UMICs as of July 2021 [25]; retrospective observational studies analysing nationally representative data; and studies with a primary focus on quantitative FRP analysis (i.e. the level, distribution and trends of CHE, impoverishment, adoption of coping strategies and FCFR).

**Fig. 2:**
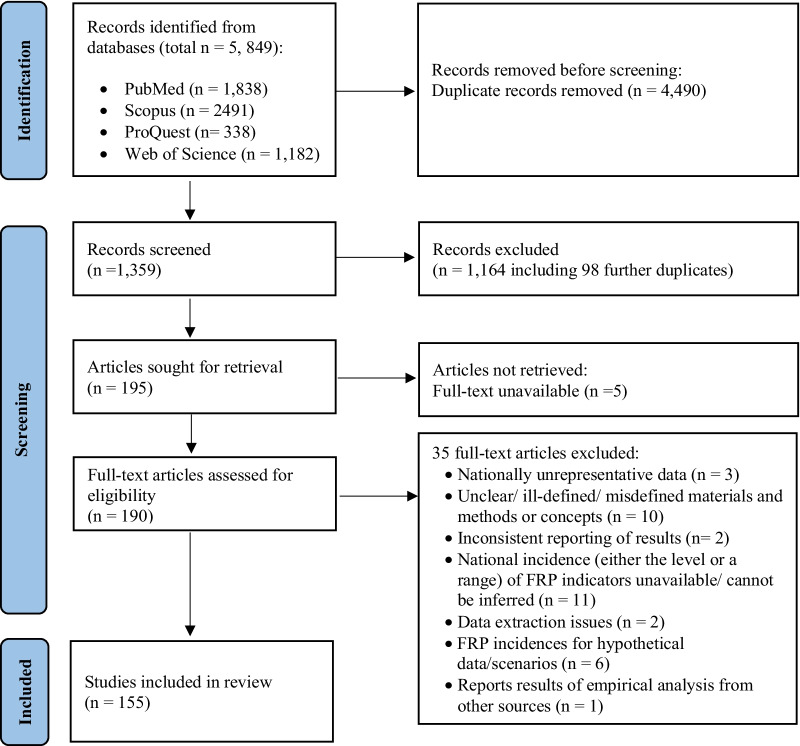
The PRISMA 2020 flow chart of the study selection process for this scoping review

The exclusion criteria were working papers; review articles; qualitative papers; analysis of OOP payments only (i.e. without any explicit analysis of the incidence of the four selected FRP indicators); modelling studies assessing FRP based on hypothetical scenarios or using assumed consumption or income instead of actual reported consumption or income; studies extracting data from nationally representative surveys but reporting the incidence of FRP indicators for a geographical subset of the population (e.g. urban only, rural only or a subset of states/provinces only). Having assessed the status of inclusion or exclusion by screening the titles and abstracts by one reviewer (TR), the selected studies for review were reassessed to confirm their eligibility. After that, full-text studies were assessed for their suitability. A second reviewer (KA) confirmed the selection based on the criteria. Figure 2 summarizes the study selection process.

### Data extraction and synthesis

The finally selected studies were analysed based on geographical focus (countries studied), source and type of data used, number of FRP indicators assessed, approaches followed to measure incidence of FRP indicators (methods and thresholds for CHE, PLs for impoverishing health expenditure, definition for coping strategies and FCFR); and rigour of analysis (intensities, distribution and trends of FRP indicators). We analysed the level of FRP by disease and condition groups (all diseases, chronic illnesses including NCDs and injuries, and communicable diseases [CDs] and maternal and perinatal conditions) by extracting the incidence of catastrophic and impoverishing OOP expenses, coping and FCFR from each publication (main text and appendix). We also examined the protective effect of health insurance from financial hardship in LMICs by examining whether insured households had lower incidence of the four FRP indicators than the noninsured (when only one year’s result was available) or if the implementation or reform of the schemes reduced the incidence of the FRP indicators over time across the whole population (when two or more years’ results were available). We reported the rank-weighted incidence of CHE when both unweighted and weighted measures were available.

## Results

### Study selection

After screening 1359 titles and abstracts, we reviewed 190 full-text peer-reviewed articles and finally included 155 articles in the study. The main reasons for exclusion were (1) national incidence (either the level or a range) of FRP indicators unavailable or could not be inferred (out of *n* = 11 studies, 9 reported odds ratios of incurring catastrophic/impoverishing OOP expenses only); (2) unclear or ill-defined or misdefined materials and methods or concepts (*n* = 10); and (3) FRP incidence for hypothetical data/scenarios (*n* = 6). The reviewed studies were quite recent, as nearly 40% of the articles were published between 2020 and mid-2021 and around 75% were published between 2018 and mid-2021 (Table 1).

**Table 1:** Distribution of the included studies by geographical coverage and data sources used (sorted by number of studies by country)

Panel A: Single-country studies (*n* = 145)								
Country	Income group, region ^a^	Total population in 2020 ^b^		Studies reviewed	Number of studies reviewed		Data source	
		Millions	%		*n*	%		
India	LwMIC, SA	1380.0	26.3	[71]; [50]; [51]; [72]; [73]; [48]; [49]; [74]; [75]; [76]; [77]; [78]; [44]; [79]; [80]; [35]; [81]; [82]; [33]; [83]; [84]; [85]; [86]; [87]; [88]; [89]; [41]; [90]; [91]; [92]; [93]; [94]; [95]; [96]; [57]	35	24.1	National Sample Survey, 1994/95, 1995/96, 1999/2000, 2004, 2004/05, 2011/12, 2014, 2017/18; Indian Human Development Survey, 2004/05, 2011/12; World Health Organization’s (WHO) Study on global AGEing and adult health (SAGE) India*, 2007/08	
China	UMIC, EAP	1402.1	26.7	[97]; [98]; [99]; [100]; [64]; [101]; [102]; [103]; [63]; [104]; [36]; [66]; [105]; [106]; [107]; [108]; [65]; [109]; [110]; [111]; [112]; [39]; [113]	23	15.9	China Health and Retirement Longitudinal Study*, 2011, 2013, 2015; National Health Services Survey, 2003, 2008, 2013; China Family Panel Studies*, 2010, 2012, 2014, 2016, 2018; China Household Finance Survey*, 2015, 2017; National Oral Health Survey, 2005; Dataset for the study ‘Evaluating Social Policy Supporting System for Vulnerable Families in Urban and Rural China’, 2014	
Iran, Islamic Rep	LwMIC, MENA	84.0	1.6	[114]; [115]; [116]; [117]; [118]; [119]; [120]; [56]; [58]; [121]	10	6.9	Households Income and Expenditure Survey, 1991, 1996, 2001, 2003 – 2017	
Ghana	LwMIC, SSA	31.1	0.6	[52]; [122]; [123]; [124]; [125]; [126]	6	4.1	Ghana Living Standard Survey, 1991/92, 1998/99, 2005/06, & 2012/13; First National Tuberculosis Patient Cost Survey, 2016	
Bangladesh	LwMIC, SA	164.7	3.1	[127]; [128]; [129]; [130]; [131]	5	3.4	Household Expenditure Survey, 1991/92, 1995/96; Household Income and Expenditure Survey, 2000, 2005, 2010, 2016; Urban Health Expenditure Survey, 2011; Bangladesh Integrated Household Survey, 2011/12	
Kenya	LwMIC, SSA	53.8	1.0	[132]; [20]; [133]; [38]; [59]	5	3.4	Kenya Household Expenditure and Utilization Survey, 2003, 2007, 2013, 2018	
Vietnam	LwMIC, EAP	97.3	1.9	[46]; [54]; [134]; [62]	4	2.8	Household Living Standards Survey, 2012, 2014, 2016; First National Tuberculosis Patient Cost Survey, 2016	
Mexico	UMIC, LAC	128.9	2.5	[12]; [135]; [61]	3	2.1	Mexican National Household Income and Expenditure Survey, 2000, 2002, 2004, 2005, 2006, 2008, & 2010; Survey of Health and Nutrition (Encuesta Nacional de Salud y Nutricion, ENSANUT), 2006, 2012	
Ethiopia	LIC, SSA	115.0	2.2	[136]; [137]; [138]	3	2.1	Household Consumption and Expenditure Survey 2010/11, 2015/16; Household Welfare Monitoring Survey 2015/16; National Tuberculosis Prevalence Survey, 2010/11	
Myanmar	LwMIC, EAP	54.4	1.0	[139]; [140]; [7]	3	2.1	World Health Survey, 2002/03; Integrated Household Living Condition Assessment, 2009/10; Poverty and Living Conditions Survey, 2015	
Nepal	LwMIC, SA	29.1	0.6	[141]; [42]; [142]	3	2.1	Living Standards Survey, 1995, 2010/11,	
Malawi	LIC, SSA	19.1	0.4	[143]; [144]; [145]	3	2.1	Integrated Household Survey, 2010/11, 2016/17	
Cambodia	LwMIC, EAP	16.7	0.3	[40]; [146]; [147]	3	2.1	Cambodian Socio-Econoamic Survey, 2004, 2009, 2014, 2015, 2016, 2017	
Mongolia	LwMIC, EAP	3.3	0.1	[148]; [43]; [149]	3	2.1	Household Socio-Economic Survey, 2012	
Indonesia	LwMIC, EAP	273.5	5.2	[150]; [151]	2	1.4	Indonesian Family Life Survey*, 1993, 1997, 2000, 2007, 2014	
Nigeria	LwMIC, SSA	206.1	3.9	[152]; [153]	2	1.4	Harmonized Nigeria Living Standard Survey, 2008/09, 2009/10	
Turkey	UMIC, ECA	84.3	1.6	[154]; [155]	2	1.4	Household Budget Surveys, 2004 – 2010, 201, 2014, 2016	
Thailand	UMIC, EAP	69.8	1.3	[156]; [55]	2	1.4	Household Socio-Economic Surveys, 1996, 1998, 2000, 2002, 2004, 2006, 2007, 2008, 2009, 2010, 2011, 2012, 2013, 2014, 2015; Health and Welfare Survey, 2015	
Colombia	UMIC, LAC	50.9	1.0	[60]; [157]	2	1.4	National Health Survey, 2007; Quality of Life National Survey, 2011	
Uganda	LIC, SSA	45.7	0.9	[158]; [159]	2	1.4	National Household Survey, 2005/06, 2009/10, 2012/13, 2016/17	
Peru	UMIC, LAC	33.0	0.6	[34]; [53]	2	1.4	National Household Survey on Living Conditions (Encuesta Nacional de Hogares, ENAHO), 2008, 2016, 2017	
Zimbabwe	LwMIC, SSA	14.9	0.3	[160]; [45]	2	1.4	National Household Survey, 2016; Health Facility-based Survey on Tuberculosis Patients, 2018	
Pakistan	LwMIC, SA	220.9	4.2	[161]	1	0.7	Household Integrated Economic Survey, 2015/16	
Brazil	UMIC, LAC	212.6	4.0	[162]	1	0.7	Brazilian Longitudinal Study of Aging (Estudo Longitudinal de Saude dos Idosos Brasileiros, ELSI) *, 2015/16	
Philippines	LwMIC, EAP	109.6	2.1	[163]	1	0.7	Family Income and Expenditure Surveys, 2000, 2003, 2006, 2009, & 2012	
South Africa	UMIC, SSA	59.3	1.1	[164]	1	0.7	Study on Global AGEing and Adult Health (SAGE) South Africa*, 2007/08	
Argentina	UMIC, LAC	45.4	0.9	[165]	1	0.7	National Survey of Household Expenditure 2012/13	
Sudan	LIC, SSA	43.8	0.8	[166]	1	0.7	National Baseline Household Survey, 2009	
Iraq	UMIC, MENA	40.2	0.8	[167]	1	0.7	Household Socio-Economic Survey, 2006/07, & 2012	
Afghanistan	LIC, SA	38.9	0.7	[168]	1	0.7	Living Conditions Survey, 2016/17	
Morocco	LwMIC, MENA	36.9	0.7	[169]	1	0.7	National Household Consumption and Expenditure Survey, 2013/14	
Zambia	LwMIC, SSA	18.4	0.3	[170]	1	0.7	Household Health Expenditure and Utilisation Survey, 2014	
Senegal	LwMIC, SSA	16.7	0.3	[171]	1	0.7	Poverty Monitoring Survey, 2011	
Tunisia	LwMIC, MENA	11.8	0.2	[172]	1	0.7	National Budget and Consumption Survey, 2000, 2005, & 2010	
Haiti	LwMIC, LAC	11.4	0.2	[32]	1	0.7	Household Living Conditions survey (Enquete sur les Conditions de Vie des Menages Apres Seisme, ECVMAS), 2012 & 2013	
Sierra Leone	LIC, SSA	8.0	0.2	[173]	1	0.7	Integrated Household Survey, 2003 & 2011	
Lao PDR	LwMIC, EAP	7.3	0.1	[174]	1	0.7	National Survey on Tuberculosis Patients, 2016	
Kyrgyz Republic	LwMIC, ECA	6.6	0.1	[175]	1	0.7	Integrated Household Surveys*, 2012 – 2018	
Liberia	LIC, SSA	5.1	0.1	[9]	1	0.7	Household Income and Expenditure Survey, 2014	
Jamaica	UMIC, LAC	3.0	0.1	[176]	1	0.7	Jamaica Survey of Living Conditions, 1996 -2002, 2004, 2006 – 2010, 2012	
Kosovo	UMIC, ECA	1.8	0.0	[177]	1	0.7	Household Budget Survey, 2014	
Eswatini (formerly Swaziland)	LwMIC, SSA	1.2	0.0	[178]	1	0.7	Household Income and Expenditure Survey, 2009/10	
Total	42 countries: LIC = 7 LwMIC = 23 UMIC = 12 EAP = 9 ECA = 3 LAC = 7 MENA = 4 SA = 5 SSA = 14	5256.6 LIC = 275.6 LwMIC = 2849.7 UMIC = 2131.3	100.0 5.3 54.2 40.5	145 studies: 2015 publications = 8 (5.5%) 2016 publications = 11 (7.6%) 2017 publications = 15 (10.3%) 2018 publications = 31 (21.4%) 2019 publications = 24 (16.6%) 2020 publications = 29 (20.0%) 2021 publications = 27 (18.6%)	145 studies: LIC = 12 LwMIC = 93 UMIC = 40	100.0 8.3 64.1 27.6	Data year range: 1991/92 – 2017/18 Latest data years: 2007/08–2009/10: 4 countries (LIC = 1, LwMIC = 2, UMIC = 1) 2010/11–2014/15: 17 countries (LIC = 2, LwMIC = 9, UMIC = 6) 2015/16–2017/18: 21 countries (LIC = 4, LwMIC = 12, UMIC = 5)	

Panel B: Multi-country studies (*n* = 10)							
Countries	Income group, region ^a^	Studies reviewed	Data sources				
122 countries	122 countries from all WB income groups, and geographic regions	[19]	Household surveys, 1984–2015				
111 countries	111 countries from all WB income groups, and geographic regions	[179]	Household surveys, 2005–2014				
Latin American countries including 15 LMICs	LwMIC, LAC (Bolivia, Haiti, Honduras, Nicaragua) UMIC, LAC (Argentina, Brazil, Colombia, Costa Rica, Dominican Republic, Ecuador, Guatemala, Mexico, Panama, Paraguay, Peru) HIC, LAC (Chile, Uruguay)	[180]	Household surveys, 1990–2013				
Bangladesh, Bhutan, India, Maldives, Nepal, Sri Lanka, Thailand, and Timor-Leste	LwMIC, EAP (Timor-Leste) LwMIC, SA (Bangladesh, Bhutan, India, Nepal, Sri Lanka) UMIC, EAP (Thailand); UMIC, SA (Maldives)	[181]	Household surveys: Bangladesh (2010), Bhutan (2012), India (2011/12), Maldives (2009), Nepal (2014), Sri Lanka (2012), Thailand (2015), and Timor-Leste (2015)				
China, Ghana, India, Mexico, the Russian Federation, and South Africa	LwMIC, SA (India) LwMIC, SSA (Ghana) UMIC, EAP (China) UMIC, LAC (Mexico) UMIC, ECA (Russian Federation) UMIC, SSA (South Africa)	[182]	World Health Organisation's Study on Global AGEing and Adult Health (WHO SAGE) * wave 2007–2010				
Bangladesh, India, Nepal, Pakistan, and Sri Lanka	LwMIC, SA (Bangladesh, India, Nepal, Pakistan, and Sri Lanka)	[183]	World Health Survey, 2002/03				
Afghanistan, Bangladesh, India, Nepal, and Pakistan	LIC, SA (Afghanistan) LwMICs, SA (Bangladesh, India, Nepal, Pakistan)	[184]	Household surveys: Afghanistan (2014), Bangladesh (2010), India (2012), Nepal (2014), and Pakistan (2014)				
Egypt, Jordan, and West Bank, and Gaza	LwMIC, MENA (Egypt, West Bank, and Gaza) UMIC, MENA (Jordan)	[185]	Household surveys: Egypt (2010/2011); Jordan (2010); West Bank and Gaza (2010)				
China, and India	LwMIC, SA (India) UMIC, EAP (China)	[186]	World Health Organisation's Study on Global AGEing and Adult Health (WHO SAGE) *, wave 2007–2010				
China, and India	LwMIC, SA (India) UMIC, EAP (China)	[187]	World Health Organisation's Study on Global AGEing and Adult Health (WHO SAGE) *, wave 2007–2010				

*LIC*  low-income country, *LwMIC*  lower-middle-income country, *UMIC*  upper-middle-income country, *LMIC*  low- and middle-income country, *HIC*  high-income country, *EAP*  East Asia and Pacific, *ECA*  Europe and Central Asia, *LAC*  Latin America and the Caribbean, *MENA*  Middle East and North Africa, *SA*  South Asia, *SSA*  sub-Saharan Africa

^a^World Bank classification of countries by income and region (https://datahelpdesk.worldbank.org/knowledgebase/articles/906519-world-bank-country-and-lending-groups)

^b^Total population data from the World Bank (https://data.worldbank.org/indicator/SP.POP.TOTL)

*Data sources marked with an asterisk represent panel data. All the other data are cross-sectional

### Study characteristics

#### Geographical coverage

Out of the 155 studies included, 145 (94.5%) were single-country studies and 10 (6.5%) were multi-country studies including two to 122 countries (Table 1). The single-country studies were on 42 LMICs from across all geographical regions, representing about 80% of the total LMIC population and 68% of the world’s population as of 2020. Consistent with large populations, the largest number of studies were on India and China, accounting for 24.1% and 15.9% of all single-country studies, respectively. Additionally, eight out of the 10 multi-country studies included India, and five consisted of both India and China. Only 8.3% (*n* = 12) of the single-country studies were on LICs, focusing on just seven countries.

### Source of data

The reviewed studies analysed data collected between 1984 and 2018. The single-country studies produced reasonably recent FRP estimates. The latest available data for 21 of the 42 countries were from 2015 or later years (Table 1). About 85% of the studies (*n* = 132) analysed cross-sectional household survey data (e.g. household income and expenditure surveys, household living standard surveys, household budget surveys). The rest (*n* = 23) used data from household panel surveys.

### FRP indicators examined

With regard to FRP indicators (Table 2), only one study (0.6%) examined all four indicators of FRP: CHE, impoverishment, coping and FCFR [7]. Most studies assessed any two indicators (49.0%), particularly CHE and impoverishment (39.4%). In total, CHE was the most frequently examined FRP indicator, with over 94% of the studies examining CHE either singly (37.4%) or in conjunction with one or more of the other three indicators (56.7%). Although impoverishment due to OOP payments is not an official UHC indicator of FRP, it was examined in half of the studies. However, the other two non-UHC indicators, coping and FCFR, were seldom recognized and analysed (in 16.8% and 3.9% of all studies, respectively).

**Table 2:** Distribution of the studies examining different FRP indicators

FRP indicators examined	Single-country studies, *n* (%)			Multi-country studies, *n* (%)	All studies, *n* (%)
	LIC	LwMIC	UMIC		
CHE only	2 (16.7)	30 (32.3)	23 (57.5)	3 (30.0)	58 (37.4)
Impoverishment only	1 (8.3)	3 (3.2)	0 (0.0)	2 (20.0)	6 (3.9)
Coping only	0 (0.0)	2 (2.2)	0 (0.0)	0 (0.0)	2 (1.3)
FCFR only	0 (0.0)	1 (1.1)	0 (0.0)	0 (0.0)	1 (0.6)
Any one indicator	3 (25.0)	36 (38.7)	23 (57.5)	5 (5.0)	67 (43.2)
CHE and impoverishment	6 (50.0)	36 (38.7)	15 (37.5)	4 (40.0)	61 (39.4)
CHE and coping	1 (8.3)	11 (11.8)	0 (0.0)	1 (10.0)	13 (8.4)
CHE and FCFR	0 (0.0)	1 (1.1)	1 (2.5)	0 (0.0)	2 (1.3)
Any two indicators	7 (58.3)	48 (51.6)	16 (40.0)	5 (50.0)	76 (49.0)
CHE, impoverishment and coping	1 (8.3)	8 (8.6)	0 (0.0)	0 (0.0)	9 (5.8)
CHE, impoverishment and FCFR	1 (8.3)	0 (0.0)	0 (0.0)	0 (0.0)	1 (0.6)
CHE, coping and FCFR	1 (0.0)	0 (0.0)	1 (2.5)	0 (0.0)	1 (0.6)
Any three indicators	2 (16.7)	8 (8.6)	1 (2.5)	0 (0.0)	11 (7.1)
All four indicators: CHE, impoverishment, coping and FCFR	0 (0.0)	1 (1.1)	0 (0.0)	0 (0.0)	1 (0.6)
Total	12 (100.0)	93 (100.0)	40 (100.0)	10 (100.0)	155 (100.0)
CHE (total)	11 (91.7)	87 (93.5)	40 (100.0)	8 (80.0)	146 (94.2)
Impoverishment (total)	9 (75.0)	48 (51.6)	15 (37.5)	6 (60.0)	78 (50.3)
Coping (total)	2 (16.7)	22 (23.7)	1 (2.5)	1 (10.0)	26 (16.8)
FCFR (total)	1 (8.3)	3 (3.2)	2 (5.0)	0 (0.0)	6 (3.9)

*FRP* financial risk protection, *CHE* catastrophic health expenditure, *FCFR* forgone care for financial reasons, *LIC* low-income country, *LwMIC* lower-middle-income country, *UMIC* upper-middle-income country

### Rigour of analysis: examination of the intensities, distribution (equity and drivers) and trends of FRP indicators

About a third of the 146 studies examining the level of CHE incidence and around half of the 78 studies measuring impoverishment incidence also examined the respective intensities in terms of catastrophic (overshoot and/or mean positive overshoot) and impoverishment gaps (normalized poverty gap and/or normalized mean poverty gap). Nearly 80% of the studies (*n* = 125) showed the distribution of either CHE or impoverishment, out of which close to half performed equity analysis in terms of distribution across household economic status (*n* = 60) and urban/rural area of residence (*n* = 62). The analysis of health systems-level drivers of financial hardship such as types of care (inpatient/outpatient) and health facilities (public/private) was available in only 16.8% and 10.4% of the studies, respectively. Drug expense-driven financial hardship was examined in just four studies [32–35].

About 38% (*n* = 58) of the included studies examined trends in FRP indicators. Eighteen of these studies, most of which were on China, analysed panel data. All but one of these studies treated each round of panel data as cross-sectional data [36]. No study took advantage of the panel data to track whether financial hardship was a short-term or long-term phenomenon (i.e. whether the same households lacked FRP over time).

### Methods used to measure FRP indicators

#### Catastrophic OOP expenditure

The distribution of studies measuring CHE by methods and thresholds applied is shown in Table 3. The reviewed studies relied solely on the three traditional methods of CHE measurement: the budget share, actual food expenditure and normative food expenditure methods [13, 14, 37]. The most frequently used was the budget share method, particularly in the LIC (81.8%), LwMIC (67.8%) and multi-country studies (100.0%). UMIC studies, on the other hand, mainly relied on the normative food expenditure (42.5%) and actual food expenditure methods (35.5%). The arguments for choosing methods, when given, were either “used in previous studies” or “established methods”. Only a quarter of the studies (*n* = 35) checked the sensitivity of CHE incidence by applying multiple methods. However, we found no strict dominance of any method in terms of the magnitude of CHE incidence. Two thirds of the studies measuring CHE used only one threshold, and the remaining applied multiple thresholds for any given method. The most frequently used threshold was 10% for the budget share method and 40% for both the actual and normative food expenditure methods.

**Table 3:** Distribution of the studies by methods and thresholds used for measuring CHE

Methods of CHE measurement adopted	Single-country studies, *n* (%)			Multi-country studies, *n* (%)	All studies, *n* (%)
	LIC	LwMIC	UMIC		
Budget share only	4 (36.4)	34 (39.1)	8 (20.0)	6 (75.0)	52 (35.6)
Actual food expenditure only	2 (18.2)	5 (5.7)	14 (35.0)	0 (0.0)	21 (14.4)
Normative food expenditure only	0 (0.0)	23 (26.4)	15 (37.5)	0 (0.0)	38 (26.0)
Any one method	6 (54.5)	62 (71.3)	37 (92.5)	6 (75.0)	111 (76.0)
Budget share and actual food expenditure	3 (27.3)	14 (16.1)	1 (2.5)	1 (12.5)	19 (13.0)
Budget share and normative food expenditure	1 (9.1)	8 (9.2)	2 (5.0)	1 (12.5)	12 (8.2)
Any two methods	4 (36.4)	22 (25.3)	3 (7.5)	2 (25.0)	31 (21.2)
Three methods: Budget share, actual food expenditure and normative food expenditure	1 (9.1)	3 (3.4)	0 (0.0)	0 (0.0)	4 (2.7)
Any method (total)	11 (100.0)	87 (100.0)	40 (100.0)	8 (100.0)	146 (100.0)
Budget share (total)	9 (81.8)	59 (67.8)	11 (27.5)	8 (100.0)	87 (59.6)
Actual food expenditure (total)	6 (54.5)	22 (25.3)	15 (37.5)	1 (12.5)	44 (30.1)
Normative food expenditure (total)	2 (18.2)	34 (39.1)	17 (42.5)	1 (12.5)	54 (37.0)
*Thresholds applied for CHE measurement*^a^					
Budget share					
10%	9 (100.0)	54 (91.5)	8 (72.7)	4 (50.0)	75 (86.2)
25%	6 (66.7)	22 (37.3)	4 (36.4)	1 (12.5)	33 (37.9)
Other	5 (55.6)	26 (44.1)	6 (54.5)	4 (50.0)	41 (47.1)
Actual food expenditure					
40%	6 (100.0)	20 (90.9)	15 (100.0)	1 (100.0)	42 (95.5)
Other	4 (66.7)	15 (68.2)	3 (20.0)	1 (100.0)	23 (52.3)
Normative food expenditure					
40%	2 (100.0)	33 (97.1)	16 (94.1)	1 (100.0)	51 (94.4)
Other	1 (50.0)	4 (11.8)	4 (23.5)	1 (100.0)	10 (18.5)

*CHE* catastrophic health expense, *LIC*  low-income country, *LwMIC*= lower-middle-income country, *UMIC*  upper-middle-income country

^a^Since some studies adopt multiple thresholds for the same method, the sum of the percentages for the different thresholds adopted for a given method is more than 100%

#### Impoverishing OOP expenditure

There were substantial variations in the type of PLs used among the 78 studies assessing impoverishing OOP expenses (Additional file 2). About 85% of these studies used only one of three types of PLs: absolute international PL (IPL) (23.1%), absolute national PL (NPL) (43.6%), relative NPL (17.9%). The remaining 15% used multiple PLs. The absolute IPL applied also varied widely, with a range of US$ 1.00–4.30 per capita per day. While all the studies examining impoverishing OOP expenses defined it as OOP expenses pushing nonpoor households into poverty, one study considered any OOP expenditure by the poor households as further impoverishing and calculated the total impoverishing effect of OOP as the sum of impoverishment and further impoverishment incidence [20].

#### Coping

The strategies for coping with healthcare expenses identified in a total of 26 studies included borrowing/loans (100.0%), sale of assets (88.5%), contributions from family and friends (26.9%), dissaving (26.9%) and other sources (3.9%). Only three studies explicitly indicated whether loans required interest payments.

#### FCFR

Among the six studies accounting for FCFR, three reported it under the heading of either health-seeking behaviour or access barriers [12, 32, 38], and the remaining three considered it as a financial burden or FRP indicator [7, 9, 39]. Each of these latter three studies defined FCFR differently: as households with ill individuals who thought their illness warranted care but decided not to seek it because of cost constraints [7]; as households experiencing a health shock, not incurring CHE, and spending less than a specified amount (in this case, US$ 10) in healthcare costs [9]; or as a pair of measures when examining inpatient care expenses through the individual level rather than household level analysis: ill individuals forgoing necessary admissions (judged by a physician) because of financial difficulties, and ill individuals taking early discharge due to financial difficulties [39].

### The evidence on FRP in LMICs

#### Illnesses, all causes

One hundred and ten studies examined at least one of the four indicators of FRP against illnesses of any kind (Additional file 3). Among these, six multi-country studies including two global studies found CHE to vary between 2% and 25% at the 10% threshold of budget share, and impoverishment to be below 5% regardless of the use of NPLs or IPLs. In particular, the rate of OOP-induced impoverishment at the US$ 1.90 per capita per day IPL was the highest (> 4%) in two LMICs among 122 countries from all income groups in the world: Bangladesh and India [19].

**Fig. 3:**
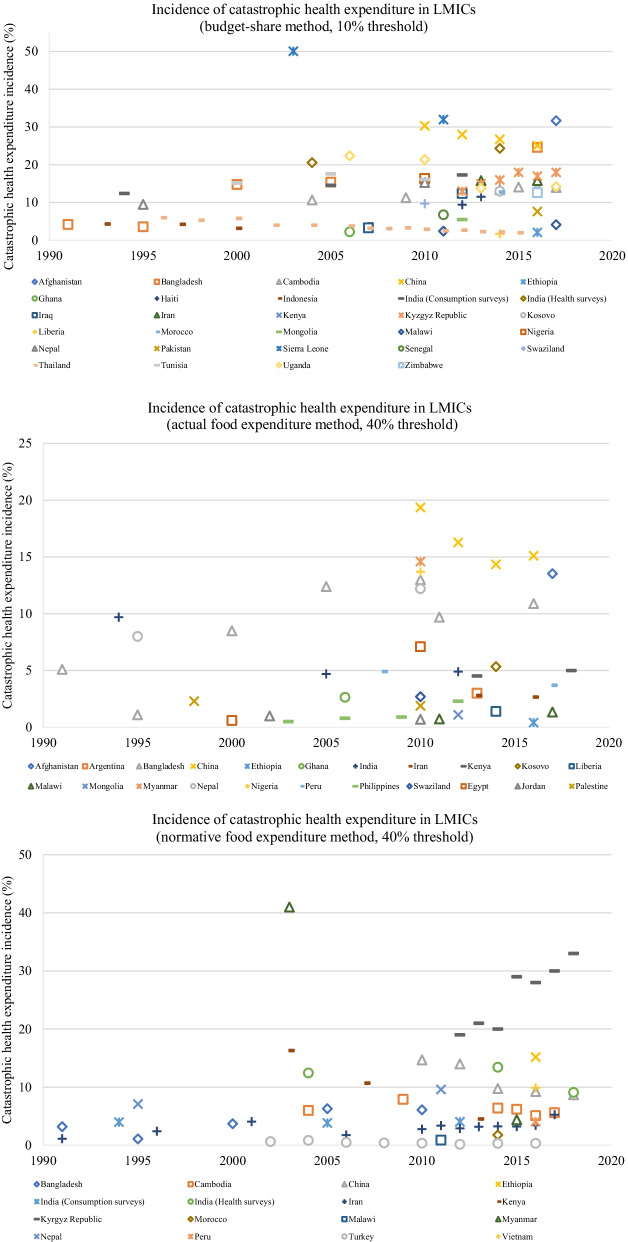
Incidence of CHE experienced due to illnesses (all causes) in LMICs. All estimates are based on national results. When more than one estimate was available across studies, only one was plotted: to show long-term changes, we considered the incidence of CHE from the study with the widest coverage of data years and filled in the incidence for additional years with figures from the other studies on the same country that had figures closer to this study. *CHE* catastrophic health expense, *LMICs*  low- and middle-income countries

Among the single-country studies, the latest national incidence of CHE at the 10% of budget share varied substantially among LICs and LwMICs, ranging from 1.8% (Liberia) to 32% (Sierra Leone), and 2.24% (Ghana) to 24.6% (Bangladesh), respectively. On the other hand, CHE in UMICs, predominantly measured using the normative food expenditure method at a 40% threshold, ranged between 0.33% (Turkey) and 8.94% (China). However, one study on China measured CHE using the 10% of budget share method and found a marginally higher CHE incidence (25.09%) than the highest in the LwMIC category mentioned earlier. Oddly, the CHE incidence in three LICs, Liberia, Malawi and Ethiopia, was very low—between 1.8% and 4.2% at 10% of budget share—whereas it was around 15% for most LMICs (Fig. 3). In particular, Liberia’s CHE incidence (1.8%) was lower than and Ethiopia’s was comparable (2.1%) to a high-performing UMIC, Thailand (2%).

The incidence of impoverishment—defined as the nonpoor households falling into poverty due to OOP expenses—at the absolute IPL of US$ 1.90 per capita per day and country-specific absolute NPLs varied in the LICs from 1.18% (Ethiopia) to 6.84% (Afghanistan) and from 0.6% (Liberia) to 4.1% (Sudan), respectively, and in the LwMICs from 0.03% (Mongolia) to 4.04% (India) and from 0.78% (Mongolia) to 8.0% (India), respectively. Unexpectedly, again, some of the poorest countries in the world, such as Liberia, Malawi and Ethiopia, had an impoverishment rate due to OOP expenses below 1% at the respective absolute NPLs. The dearth of national-level UMIC studies reporting the incidence of impoverishment (*n* = 6) and the differences in the NPLs and IPLs applied in their measurement restricted meaningful intra- and inter-country income group comparisons.

In terms of coping strategies, borrowing for healthcare expenses was the lowest for Cambodia (2.2–2.5%) and the highest for India (4.3–41.4%) [40, 41]. National incidence for FCFR was available for only three countries: 3.67% in Myanmar, 8% in Liberia and 13.4% in Haiti [7, 9, 32].

#### Chronic diseases

Thirty-eight studies reported the incidence of FRP indicators related to chronic diseases including NCDs and/or injuries (Additional file 4). With no studies on any LIC, the single-country studies (*n* = 35/38) were dominated by India (*n* = 11) and China (*n* = 10). Only 15 studies reported national results, in which CHE incidence at the 10% of budget share, 40% of nonfood expenditure and 40% of food-based non-subsistence expenditure ranged from 9.6% (Nepal) to 39.7% (Bangladesh), 3.3% (Mongolia) to 17.8% (Bangladesh), and 1.5% (Nepal) to 33.81% (five South Asian LwMICs), respectively. The national incidence of impoverishment, available in just four studies, was between 1.25% (Nepal) and 7.9% (Mongolia) at the country-specific absolute NPL [42, 43]. The few individual disease-specific FRP studies (*n* = 10) were conducted in China, India and Nepal only. These studies mainly dealt with diabetes, cancers and heart disease, which caused 0–17%, 7.6–58% and 26.8–63.8% of households to incur CHE, respectively. Coping for chronic diseases was mainly reported for India (*n* = 8/10), where cancer treatment-related hospitalization led to the highest proportion of households (40–50%) borrowing money or selling assets, or seeking contributions from friends and family [44]. No study examined FCFR for chronic diseases.

### CDs and maternal and perinatal conditions

FRP studies on CDs (*n* = 13) were mostly concerned with tuberculosis (TB) in LwMICs (*n* = 7) (Additional file 5). CHE due to TB could be as high as 80% (Zimbabwe) despite the availability of free treatments in public facilities [45]. Drug-resistant TB was responsible for about 14% to 65% higher burden than the drug-susceptible variety [45, 46]. Note that, in line with WHO’s End TB Strategy to end the global TB epidemic, most TB studies defined OOP expenses as the sum of direct medical, nonmedical and indirect expenditures for seeking TB care [47]. Therefore, one should be cautious when comparing CHE incidence due to TB and other diseases, including any chronic illness. TB treatments pushed at least 14.2% of households into poverty and forced about 5.1–51.5% of households to adopt coping strategies. Notably, no study on FRP from CDs was available for any UMIC.

Care for health conditions, such as childbirth at health facilities in LwMICs, particularly in India, caused at least 20% of households to incur CHE (Additional file 6) [48, 49]. The incidence was two to three times higher when OOP expenses for antenatal, delivery and postnatal care were considered together [50, 51].

### FRP through health insurance

The results from examining the protective effect of different health insurance schemes (Additional file 7) suggest that health insurance in LMICs did not consistently offer a higher degree of FRP. Even though insurance lowered CHE in six studies (in Ghana, Iran, Peru, Thailand and Viet Nam) [34, 52–56], three studies reported higher CHE in India, Iran and Kenya [57–59], and another three studies reported not necessarily lower CHE in Colombia, Mexico and Vietnam [60–62]. Again, two of these studies showed mixed results: insured households incurred higher CHE but lower impoverishment than the noninsured in India [57], and CHE decreased but impoverishment increased just after launching the Health Transformation Plan in Iran [56]. Notably, although both Thailand and China brought nearly all people under health insurance coverage (100% and 97%, respectively), the levels of FRP in these countries were quite different. Thailand achieved exemplary low levels of CHE (2% at 10% of budget share) and impoverishment (0.07% at US$ 1.90 per capita per day) by implementing its public health insurance schemes [55]. In comparison, the incidence of CHE and impoverishment for the insured households was quite high in China (about 15% at 40% of non-subsistence expenditure, and 1.3–7.6% at the relative NPL, respectively) [63, 64]. Subgroup studies also did not confirm a protective effect of insurance. However, from the scant number of studies reporting coping strategies (*n* = 2) and FCFR (*n* = 2), insurance seemed to slash the need to adopt coping strategies to some degree and removed the financial barriers to accessing healthcare, even though it did not entirely eliminate them [12, 39, 52].

## Discussion

This scoping review summarizes the most recent empirical evidence on FRP against the OOP mode of payment for healthcare in LMICs. Compared with the previous relevant review by Alam and Mahal, the volume of LMIC studies has grown substantially since 2015 [24]. The UN’s adoption of the SDG in 2015, which includes FRP as a crucial component of the UHC target (SDG 3.8.2), most likely boosted the literature growth. However, there are significant research gaps.

The country coverage of the LMIC literature was biased towards India and China. Notably, there was a complete absence of studies on FRP from chronic NCDs or injuries in any LIC and CDs in any UMIC. Only one study portrayed a comprehensive picture of FRP in a country (Myanmar) by examining all four indicators of FRP as outlined in our conceptual framework: CHE, impoverishment, coping and FCFR. The LMIC literature conceptualized (the lack of) FRP narrowly, quantifying it mainly through CHE incidence and less frequently through impoverishment incidence. Just a handful of studies identified and measured the adoption of coping strategies and FCFR as indicators of a lack of FRP. Moreover, in measuring CHE, the studies invariably used traditional methods (with the majority placing arguments such as “used in previous studies” or “established methods”), despite their limitations in accurately representing financial catastrophe across households of different economic status. Furthermore, the health systems-level drivers of financial hardship, such as type of service (drugs, diagnosis, etc.), type of care (inpatient/outpatient) and source of care (public/private), were examined in a limited number of studies.

From the available studies, we found FRP to vary across countries. With the variation being substantial among the LICs, there was no discernible pattern in the level of protection amongst the country income categories. Contrary to general expectations, we found three of the poorest countries in the world (Liberia, Ethiopia and Malawi) to have a very low incidence of impoverishment and CHE, comparable to or even lower than some high-performing UMICs. Both CDs and chronic NCDs caused very high CHE, but the respective magnitudes could not be compared because of the differences in measurement methods.

The evidence regarding the protective effect of health insurance in LMICs is mixed in reducing CHE and impoverishment but positive (albeit from a minimal number of studies) in curtailing the need to adopt coping strategies and forgoing needed healthcare for financial reasons. The uneven success in reducing CHE and impoverishment may be due to country-specific variations in the implemented health insurance schemes regarding their type (public, private), service coverage, consumer cost-sharing and provider reimbursement mechanisms, among other factors. Thailand’s exemplary record in reducing CHE and impoverishment is credited to the country’s implementation of general tax-funded public health insurance schemes covering all people and offering a comprehensive benefits package with no co-payment at the point of service [55]. Also vital were health technology assessments to identify cost-effective interventions to add to the benefits package and closed-end provider payments (capitation for outpatient services and diagnosis-related groups under the global budget for inpatient services) [55]. Conversely, the ungenerous benefits package and fee-for-service payment system were the potential factors explaining relatively high CHE and impoverishment (i.e. low FRP) in China despite near-universal insurance coverage [65, 66].

The low incidence of CHE and impoverishment in some of the LICs does not necessarily mean a high level of FRP in those countries. The way these two indicators are defined does not count all the households that lack financial protection. First, the conventional measurement of impoverishment incidence considers only nonpoor households that become poor due to healthcare spending. Therefore, the large number of households already living in extreme poverty in these three LICs (Malawi 69.2%, Ethiopia 30.8%, Liberia 44.4% at US$ 1.90 per capita per day) whose poverty condition may have been aggravated by healthcare expense (i.e. the further impoverished households) were not included in the impoverishment incidence figure [67]. Notably, one LwMIC study we reviewed estimated that the total poverty effect of OOP expenses in Kenya would be about 40 times higher (39.14% instead of 1.02%) if the incidence of further impoverishment (38.12%) were accounted for [20]. The second reason, which explains the low incidence of both impoverishment and CHE, is that these indicators show the effect of seeking healthcare and paying for it. Unless a study assessing FRP includes forgone care or unmet need analysis, it is difficult to determine whether low CHE and impoverishment incidences reflect inadequate service access or high FRP. Poor access to services prevents people from seeking care, resulting in zero OOP payments for their families. These households are not counted as incurring catastrophic or impoverishing OOP expenses, resulting in low incidence of these indicators [68]. The barriers to accessing healthcare could be purely nonfinancial (e.g. unavailable, inaccessible or unacceptable services). In that case, health systems with low CHE and impoverishment may reflect a high degree of FRP (but inadequate access to healthcare). Alternatively, if financial reasons (e.g. affordability) prevent healthcare utilization (i.e. the incidence of FCFR is positive), the CHE and impoverishment incidences underestimate the population lacking FRP. However, it is also essential to recognize that if healthcare is widely accessible for free or at a nominal cost (e.g. insurance-covered care, community-based care, public or donor-funded healthcare), OOP expenses would be zero or sufficiently small to result in low incidence of CHE and impoverishment, reflecting a high degree of FRP (and high access to care).

Central to the SDG agenda is to “leave no one behind”. Likewise, the UHC target specifies that no one should face undue financial hardship to access healthcare. Therefore, identifying further impoverished households and households unable to afford healthcare aligns with the SDG spirit and, needless to say, is crucial to guiding where policies should specifically focus to improve FRP.

Similarly, using methods to measure CHE that do not precisely assess the true incidence across households of different economic status has implications for tracking progress towards achieving UHC, particularly for assessing inequalities within and across countries, which is one of the core concerns of the SDG [15]. The studies in this review, particularly those on LICs and LwMICs, predominantly used the budget share method to measure CHE. The method’s application of constant thresholds ignores that poorer households may react differently to allocating the same fraction of household budget than their wealthier counterparts. Hence, the resulting CHE incidence is likely to be underestimated (and FRP overestimated) for the poor, for whom even minimal healthcare expense can be disastrous.

Even though the advancement of methods for measuring FRP suggests a way forward, the literature shows that LMIC studies have not taken advantage of it. In particular, the normative food, housing (rent) and utilities method considers any healthcare expense of the poor households as catastrophic [18]. Unlike other CHE measurement techniques, this method can achieve an effective threshold that rises with household economic status and thus measures CHE more precisely [15]. Additionally, it highlights three segments of the population that are rarely visible in FRP studies using the traditional methods: households that are further impoverished because of healthcare payments, households that are at risk of impoverishment due to healthcare expenses, and households that do not spend on healthcare, some of whom might have unmet needs due to cost, distance or other barriers [18]. Such a way of assessing FRP yields actionable evidence for policy and promotes pro-poor policies to break the cycle between ill health and poverty [4].

On another note, concerns have been raised about the usefulness of the FRP indicator trends in understanding whether the same households remain trapped in poverty for a long period or whether impoverishment is just a one-time event, which can only be examined if the same households can be tracked over time or if there are frequent panel data [3, 8]. Unfortunately, 18 out of the 23 studies measuring FRP from panel data used more than one round of data (all but one study treated each round of data as cross-sectional), but none examined whether the poverty induced by healthcare was transient or permanent.

Based on the gaps in the literature we have reviewed, we recommend future research on FRP in LMICs to systematically address the gaps in country coverage and disease coverage, and to measure FRP comprehensively using indicators that offer information on the full economic impact of requiring OOP payment for healthcare, while paying specific attention to the use of methods that address the limitations of the traditional techniques for measuring CHE and impoverishment.

Future studies need to place greater emphasis on LICs, particularly in countries where no recent study is available. Even though large global-level studies cover many of the LICs that have no country-specific studies, such studies rarely address the questions of distribution and underlying drivers. Filling the knowledge gap regarding FRP against chronic diseases in LICs is also essential, as SDG 3.4.1—the probability of dying between age 30 and 70 due to leading NCDs (cardiovascular diseases, cancer, diabetes or chronic respiratory diseases)—is the highest in LICs across the globe [69]. Further research is also needed in all LMICs to understand the disease-specific FRP against the NCDs that pose substantial disease burden. Besides cancer, diabetes and cardiovascular diseases, these include mental disorders and respiratory, digestive and genitourinary diseases. Given the recent finding that average treatment costs are higher for respiratory diseases than for cancer, diabetes or cardiovascular diseases in LMICs, FRP against respiratory diseases should be a research priority [70].

Failure to accurately and comprehensively measure the level and pattern of financial protection will result in misguided policy responses. Therefore, to track progress towards UHC in the SDG, we suggest presenting CHE estimates applying all CTP methods, including the normative food, housing (rent) and utilities technique, besides the budget share method. Such studies are expected to facilitate informed decision-making and to prevent potential political manipulation in demonstrating the success or failure of a policy. Presenting CHE measures using all available methods is also warranted given that our study did not find any clear dominance of the magnitude of CHE incidence across methods. We also recommend that future financial protection studies supplement the CHE and impoverishment incidences with coping and FCFR to obtain a comprehensive picture and, as such, valuable insights into the implications of the need to pay for healthcare from OOP on household welfare. It is also necessary to examine context-specific details of the healthcare systems’ components, such as the type of health insurance scheme, its costs and coverage of services, to better understand the mixed results of health insurance protection, and access to healthcare (financial vs nonfinancial barriers to access, community vs hospital-based provision of healthcare, etc.) to make sense of the low incidence of CHE and impoverishment in LICs. Additionally, countries like China should take advantage of their wealth of different longitudinal databases to determine whether the households falling into poverty (or facing CHE) due to healthcare expenses bounce back shortly after or remain wedged in there for an extended period. Such data sources can also be used to validate the hypotheses about the long-term consequences of adopting coping strategies and consequent development of disease and economic impacts associated with forgone treatment.

The main limitation of this review is that it did not include grey literature or studies published in a language other than English. Therefore, we may have missed some potentially relevant national studies which could have broadened the knowledge base.

## Conclusion

In this study, we critically reviewed 155 studies published between 2015 and mid-2021 on FRP in healthcare in LMICs. Despite the incredible growth in the literature since 2015 which uses reasonably recent data, major gaps exist. First, while there is at least some research on FRP in every region containing LMICs, there are few studies on LICs. Additionally, the large volume of studies on LwMICs and UMICs mainly focus on India and China, respectively. Second, there is no FRP study on chronic illness in any LIC, and no study on CDs in any UMIC. Also, LMICs of all income categories lack individual disease-specific studies, particularly those that impose high treatment costs and disease burden (such as respiratory diseases). Third, there are gaps in utilizing available data to gain more insight into the drivers and duration of financial hardship. Health systems-level drivers of financial hardship such as types of service, care and health facilities are examined in a limited number of studies. Next, particularly for China, even though panel data were frequently available where the surveyed households could be tracked over the years, there was no attempt to determine whether financial hardship due to OOP health expenses were a one-time or long-term incident for a household. Fourth, the FRP literature in LMICs conceptualizes FRP narrowly. Only one study measures FRP comprehensively by examining all four indicators (CHE, impoverishment, coping and FCFR), each of which addresses a different aspect of FRP. Most studies measure FRP by examining either both CHE and impoverishment (39.4%) or CHE alone (37.4%). Fifth, the studies measuring CHE and impoverishment invariably depend on the traditional measurement methods despite their limitations in accurately representing FRP across households of different economic status. In general, the LMIC literature has not kept pace with the recent development of methodologies that address the limitations of earlier methods. Because of the lack of comprehensive measurement of FRP indicators, coupled with the use of dated measurement methods, the literature is unable to provide a reliable representation of the actual level of protection enjoyed by the households in LMICs.

Disregarding the methodological issues, the available estimates of FRP against illness vary substantially among the countries, with no distinct pattern in the level of protection amongst the country income categories. From the latest available national estimates, the incidence of CHE (any method), impoverishment, coping and FCFR due to illness (all causes) in LMICs ranges from 0.33% to 32%, 0.03% to 8%, 2.2% to 41.4%, and 3.7% to 13.7%, respectively. Surprisingly, some of the poorest countries in the world have very low incidence of CHE and impoverishment, even lower than or comparable to that in Thailand, a high-performing UMIC. LMIC households affected by chronic illness or infectious diseases such as TB experience very high incidence of CHE, ranging from 1.5% to 39.7% and 0.0% to 80.0%, respectively. However, it was not possible to ascertain which of these two broad disease groups caused higher CHE incidence because of the differences in measurement methods. Although it might seem obvious that households with formal health insurance will enjoy greater FRP than those without, the reviewed studies indicate that this is not always the case in LMICs.

Future research on FRP in LMICs should thoroughly address the gaps in country coverage and disease coverage, and measure FRP comprehensively using a broader range of indicators that offer information on the full economic impact of OOP health spending, while paying specific attention to the use of methods that address the limitations of the traditional techniques for measuring CHE and impoverishment. Where possible, longitudinal data should be employed to study the long-term consequences of OOP payments for healthcare.

## Supplementary Information

**Additional file 1.** Sample database search string. Search string for PubMed.

**Additional file 2.** Distribution of studies by poverty lines used in impoverishment incidence measurement. Poverty lines used in impoverishment incidence measurement categorized across single-country and multi-country studies.

**Additional file 3.** Financial risk protection against all illnesses. The studies on financial risk protection against all illnesses are summarized by author(s) name and year, country, data source, incidences of catastrophic health expenditure, impoverishment, coping, and forgone care for financial reasons.

**Additional file 4.** Financial risk protection against chronic diseases including noncommunicable diseases and injuries. The studies on financial risk protection against chronic diseases including noncommunicable diseases and injuries are summarized by author(s) name and year, country, data source, disease (study subgroup), incidences of catastrophic health expenditure, impoverishment, and coping.

**Additional file 5.** Financial risk protection against communicable and infectious diseases. The studies on financial risk protection against communicable and infectious diseases are summarized by author(s) name and year, country, data source, disease(s)/condition(s), incidences of catastrophic health expenditure, impoverishment, and coping.

**Additional file 6.** Financial risk protection against other conditions. The studies on financial risk protection against other conditions are summarized by author(s) name and year, country, data source, conditions, incidences of catastrophic health expenditure, impoverishment, and coping.

**Additional file 7.** Financial risk protection through insurance and other schemes. The studies on financial risk protection through insurance and other schemes are summarized by author(s) name and year, country, data source, intervention/financial protection scheme examined, disease(s)/condition(s) (subgroup) incidences of catastrophic health expenditure, impoverishment, coping, and forgone care for financial reasons.

## Data Availability

All data analysed during this study are included in this article and its supplementary information files.

## Abbreviations

CD: Communicable disease
CHE: Catastrophic health expenditure
EAP: East Asia and Pacific
ECA: Europe and Central Asia
FCFR: Forgone care for financial reasons
FRP: Financial risk protection
IPL: International poverty line
LAC: Latin America and the Caribbean
LIC: Low-income country
LMIC: Low- and middle-income countries
LwMIC: Lower-middle-income country
MENA: Middle East and North Africa
NCD: Noncommunicable disease
NPL: National poverty line
OOP: Out-of-pocket
PL: Poverty line
PRISMA: Preferred Reporting Items for Systematic Reviews and Meta-Analysis
SDG: Sustainable Development Goals
SSA: Sub-Saharan Africa
TB: Tuberculosis
UHC: Universal health coverage
UMIC: Upper-middle-income country
UN: United Nations
US$: United States dollar
WB: World Bank
