# Supplementary material for: Financial risk protection from out-of-pocket health spending in low- and middle-income countries: a scoping review of the literature
Source: Health Res Policy Syst. 2022 Jul 29;20:83. doi: 10.1186/s12961-022-00886-3 (PMC9336110; doi:10.1186/s12961-022-00886-3)
Supplement: Supplementary file 1 — Additional file 1. Sample database search string. Search string for PubMed. [file 12961_2022_886_MOESM1_ESM.docx]

**Database: PubMed**

**Search string**

(("health expenditures"[MeSH] "out of pocket" [tw] "out-of-pocket" [tw] OR "out of pocket payment*" [tw] OR "out of pocket expenditure*" [tw] OR "out of pocket expens*" [tw] OR "out of pocket spending*" [tw] OR "out of pocket cost*" [tw] OR "out-of-pocket payment*" [tw] OR "out-of-pocket expenditure*" [tw] OR "out-of-pocket expens*" [tw] OR "out-of-pocket spending*" [tw] OR "out-of-pocket cost*" [tw] OR "health* payment*" [tw] OR "health* expenditure*" [tw] OR "health* expense*" [tw] OR "health* spending*" [tw] OR "health care cost*" [tw] OR "health care payment*" [tw]OR "health care expenditure*" [tw] OR "health care expense*" [tw] OR "health care spending*" [tw] OR "medical expenditure*" [tw]OR "medical expense*" [tw] OR "medical spending*" [tw] OR "medical cost*" [tw] OR "cost sharing" [tw] OR "cost-sharing" [tw] OR "patient payment*" [tw] OR "co-payment*" [tw] OR copayment* [tw] OR "user fee*" [tw] OR "user charge*" [tw]) AND ("universal health care"[MeSH Terms] OR "universal health coverage"[Text Word] OR "Catastrophic Illness"[MeSH Terms] OR "financial stress*"[MeSH Terms] OR "poverty"[MeSH Terms] OR "catastroph*"[Text Word] OR "impoverish*"[Text Word] OR "financial protection"[Text Word] OR "financial risk protection"[Text Word] OR "financial hardship*"[Text Word] OR "financial burden*"[Text Word] OR "financial challeng*"[Text Word] OR "financial pressure*"[Text Word] OR "economic burden*"[Text Word] OR "economic hardship*"[Text Word] OR "health shock*"[Text Word] OR ("coping"[Text Word] OR "cope"[Text Word] OR "coping strateg*"[Text Word] OR "distress financ*"[Text Word] OR "hardship financ*"[Text Word] OR "consumption smoothing"[Text Word] OR "income smoothing"[Text Word] OR "consumption insurance"[Text Word]) OR ("cost-related unmet need"[Text Word] OR ("financial barrier"[Text Word] AND "unmet need"[Text Word]) OR (("forgo care"[Text Word] OR "forgo health care"[Text Word] OR "forgo healthcare"[Text Word] OR "forgone care"[All Fields] OR "forgone health care"[Text Word] OR "forgone healthcare"[Text Word]) AND ("cost"[Text Word] OR "economic reason*"[Text Word] OR "financial reason*"[Text Word] OR "unaffordable"[Text Word]))))) AND (((Afghanistan [tw] OR "Burkina Faso" [tw] OR Burundi [tw] OR "Central African Republic" [tw] OR "Central Africa" [tw] OR Chad [tw] OR "Congo" [tw] OR Eritrea [tw] OR Ethiopia [tw] OR Gambia [tw] OR Guinea [tw] OR "Guinea-Bissau" [tw] OR "North Korea" [tw] OR Liberia [tw] OR Madagascar [tw] OR Malawi [tw] OR Mali [tw] OR Mozambique [tw] OR Niger [tw] OR Rwanda [tw] OR "Sierra Leone" [tw] OR Somalia [tw] OR "South Sudan" [tw] OR Sudan [tw] OR Syria [tw] OR "Syrian Arab Republic" [tw] OR Togo [tw] OR Uganda [tw] OR Yemen) OR (Algeria [tw] OR Angola [tw] OR Bangladesh [tw] OR Belize [tw] OR Benin [tw] OR Bhutan [tw] OR Bolivia [tw] OR "Cabo Verde" [tw] OR Cambodia [tw] OR Cameroon [tw] OR Comoros [tw] OR Congo [tw] OR "Côte d'Ivoire" [tw] OR Djibouti [tw] OR Egypt [tw] OR "El Salvador" [tw] OR Eswatini [tw] OR Ghana [tw] OR Haiti [tw] OR Honduras [tw] OR India [tw] OR Indonesia [tw] OR "Iran" [tw] OR Kenya [tw] OR Kiribati [tw] OR "Kyrgyz Republic" [tw] OR "Lao PDR" [tw] OR Lesotho [tw] OR "Timor-Leste" [tw] OR Mauritania [tw] OR Micronesia [tw] OR Mongolia [tw] OR Morocco [tw] OR Myanmar [tw] OR Nepal [tw] OR Nicaragua [tw] OR Nigeria [tw] OR Pakistan [tw] OR "Papua New Guinea" [tw] OR Philippines [tw] OR Samoa [tw] OR "São Tomé and Principe" [tw] OR Senegal [tw] OR "Solomon Islands" [tw] OR "Sri Lanka" [tw] OR Tajikistan [tw] OR Tanzania [tw] OR Tunisia [tw] OR Ukraine [tw] OR Uzbekistan [tw] OR Vanuatu [tw] OR Vietnam [tw] OR "West Bank and Gaza" [tw] OR Zambia [tw] OR Zimbabwe)) OR (Albania OR "American Samoa" [tw] OR Argentina [tw] OR Armenia [tw] OR Azerbaijan [tw] OR Belarus [tw] OR "Bosnia and Herzegovina" [tw] OR Botswana [tw] OR Brazil [tw] OR Bulgaria [tw] OR China [tw] OR Colombia [tw] OR "Costa Rica" [tw] OR Cuba [tw] OR Dominica [tw] OR "Dominican Republic" [tw] OR Ecuador [tw] OR "Equatorial Guinea" [tw] OR Fiji [tw] OR Gabon [tw] OR Georgia [tw] OR Grenada [tw] OR Guatemala [tw] OR Guyana [tw] OR Iraq [tw] OR Jamaica [tw] OR Jordan [tw] OR Kazakhstan [tw] OR Kosovo [tw] OR "St. Lucia" [tw] OR Lebanon [tw] OR Libya [tw] OR Malaysia [tw] OR Maldives [tw] OR "Marshall Islands" [tw] OR Mauritius [tw] OR Mexico OR Moldova [tw] OR Montenegro [tw] OR Namibia [tw] OR "North Macedonia" [tw] OR Panama [tw] OR Paraguay [tw] OR Peru [tw] OR Romania [tw] OR "Russian Federation" [tw] OR Serbia [tw] OR "South Africa" [tw] OR "St. Vincent and the Grenadines" [tw] OR Suriname [tw] OR Thailand [tw] OR Tonga [tw] OR Turkey [tw] OR Turkmenistan [tw] OR Tuvalu)) Filters: English, from 2015 - 2021
