# Supplementary material for: Financial risk protection from out-of-pocket health spending in low- and middle-income countries: a scoping review of the literature
Source: Health Res Policy Syst. 2022 Jul 29;20:83. doi: 10.1186/s12961-022-00886-3 (PMC9336110; doi:10.1186/s12961-022-00886-3)
Supplement: Supplementary file 2 — Additional file 2. Distribution of studies by poverty lines used in impoverishment incidence measurement. Poverty lines used in impoverishment incidence measurement categorized across single-country and multi-country studies. [file 12961_2022_886_MOESM2_ESM.docx]

**Additional file 2:** Distribution of studies by poverty lines used in impoverishment incidence measurement

| Poverty lines | Single-country studies, n (%) | | | Multi-country studies, n (%) | All studies, n (%) |
| --- | --- | --- | --- | --- | --- |
|  | LIC | LwMIC | UMIC |  |  |
| Absolute IPL only | 2 (22.2) | 9 (18.8) | 4 (26.7) | 3 (50) | 18 (23.1) |
| Absolute NPL only | 5 (55.6) | 26 (54.2) | 3 (20.0) | 0 (0.0) | 34 (43.6) |
| Relative NPL only | 0 (0.0) | 7 (14.6) | 5 (33.3) | 2 (33.3) | 14 (17.9) |
| Any one PL | 7 (77.8) | 42 (87.5) | 12 (80.0) | 5 (83.3) | 66 (84.6) |
|  |  |  |  |  |  |
| Absolute IPL and absolute NPL | 2 (22.2) | 3 (6.3) | 1 (6.7) | 0 (0.0) | 6 (7.7) |
| Absolute IPL and relative NPL | 0 (0.0) | 0 (0.0) | 0 (0.0) | 1 (16.7) | 1 (1.3) |
| Absolute NPL and relative NPL | 0 (0.0) | 1 (2.1) | 0 (0.0) | 0 (0.0) | 1 (1.3) |
| Any two PL | 2 (22.2) | 4 (8.3) | 1 (6.7) | 1 (16.7) | 8 (10.3) |
|  |  |  |  |  |  |
| Three PLs: Absolute IPL, absolute NPL, and relative NPL | 0 (0.0) | 0 (0.0) | 1 (6.7) | 0 (0.0) | 1 (1.3) |
| Not specified/ not available | 0 (0.0) | 2 (4.2) | 1 (6.7) | 0 (0.0) | 3 (3.8) |
|  |  |  |  |  |  |
| Any PL (total) | 9 (100.0) | 48 (100.0) | 15 (100.0) | 6 (100.0) | 78 (100.0) |
|  |  |  |  |  |  |
| Absolute IPL (total) | 4 (44.4) | 12 (25.0) | 6 (40.0) | 4 (66.7) | 26 (33.3) |
| Absolute NPL (total) | 7 (77.8) | 30 (62.5) | 5 (33.3) | 0 (0.0) | 42 (53.8) |
| Relative NPL (total) | 0 (0.0) | 8 (16.7) | 6 (40.0) | 3 (50.0) | 17 (21.8) |
| Any NPL (total) | 7 (77.8) | 37 (77.1) | 10 (66.7) | 3 (50.0) | 57 (73.1) |

LIC = low-income country, LwMIC = lower middle-income country, UMIC = upper middle-income country

IPL = International poverty line, ANPL = Absolute national poverty line, RNPL = Relative national poverty line

Note: Since some studies adopt multiple poverty lines, the sum of the percentages for the different poverty lines applied for a given type of study (single- or multi-country) is more than 100%
