# Supplementary material for: Financial risk protection from out-of-pocket health spending in low- and middle-income countries: a scoping review of the literature
Source: Health Res Policy Syst. 2022 Jul 29;20:83. doi: 10.1186/s12961-022-00886-3 (PMC9336110; doi:10.1186/s12961-022-00886-3)
Supplement: Supplementary file 3 — Additional file 3. Financial risk protection against all illnesses. The studies on financial risk protection against all illnesses are summarized by author(s) name and year, country, data source, incidences of catastrophic health expenditure, impoverishment, coping, and forgone care for financial reasons. [file 12961_2022_886_MOESM3_ESM.docx]

**Additional file 3**: Financial risk protection against illness, all causes

| **Study** | **Country** | **Data Source** | **Incidence of CHE (%)** | **Incidence of Impoverishment (% point)** | **Incidence of Coping (%)** | **Incidence of Forgone Care for Financial Reasons (%)** |
| --- | --- | --- | --- | --- | --- | --- |
| **National results: multi-country studies (n = 6)** | | | | | | |
| Wagstaff & Neelsen, 2020 | 111 countries from all income groups | Household surveys 2005 - 2014 | approx. 2 - 25 (TE_Cata10) | n/a | n/a | n/a |
| Wang et al., 2018 | eight countries in the WHO South-East Asia Region | Household surveys, 2009 - 2015 | 1.88 - 19.88 (TE_Cata10); 0.36 - 6.17 (TE_Cata25) | 0.00 - 4.21 (IPL US$ 1.9/capita/day); <0.01 - 4.56 (IPL US$ 3.1/capita/day) | n/a | n/a |
| Wagstaff et al., 2018 | 122 countries from all income groups | Household surveys 1984 - 2015 | n/a | > 4.0 in Bangladesh and India; the highest among all countries (IPL US$ 1.9/ capita/day) | n/a | n/a |
| Rahman et al., 2017 | five South Asian countries | Household surveys, 2010 - 2014 | 4.4 - 17.9 (TE_Cata10) | 1.4 - 4.9 (RNPL) | n/a | n/a |
| Wagstaff et al., 2015 | 17 Latin American countries | Household surveys 1990 - 2014 | approx. < 1 - 12.5 (TE_Cata25) | approx. 0.0 pp - 5.0 (IPL US$ 2/capita/day) | n/a | n/a |
| Rashad & Sharaf, 2015 | three Arab countries: Egypt, Jordan, and Palestine | Household surveys 2010, 2010/11 | 2.7 - 22.4 (TE_Cata10); 0.4 - 3.4 (TE_Cata25); 0.7 - 7.1 (nFE_Cata40) | 0.4 (IPL US$ 1.9/capita/day); 2.7 (IPL US$ 3.1/capita/day) (reported for one country (Egypt) only | n/a | n/a |
| **National results single-country studies: low- income counties (n = 11)** | | | | | | |
| Dastan et al., 2021 | Afghanistan | Living Conditions Survey data for 2016-2017 | 31.69 (TE_Cata10); 11.61 (TE_Cata25); 13.53 (nFE_Cata40) | 6.84 (IPL US$ 1.9/ capita/day); 6.53 (IPL US$ 3.2/ capita/day) | saving: 23 - 33, loan/ borrowing: 26-39; sale of assets: 7-10; other sources: 6-10 | n/a |
| Mulaga et al., 2021 | Malawi | Integrated Household Survey (IHS), 2016/17 | 4.14 (TE_Cata10); 0.84 (TE_Cata25); 1.34 (nFE_Cata40) | 1.60 (ANPL) | n/a | n/a |
| Kiros et al., 2020 | Ethiopia | Household Consumption and Expenditure (HCE) and Welfare Monitoring (WM) Surveys 2015/16 | 2.1 (TE_Cata10); 0.05 (TE_Cata25); 0.41 (nFE_Cata40); 15.18 (CTP_PL_Cata40) | 0.9 (ANPL) | n/a | n/a |
| Kwesiga et al., 2020 | Uganda | National Household Surveys, 2005/06, 2009/10, 2012/13, & 2016/17 | 14.2 (TE_Cata10); 2.7 (TE_Cata25) | 5.2 (IPL US$ 1.90/ capita/day); 2.7 (ANPL) | n/a | n/a |
| Obse & Ataguba, 2020 | Ethiopia | Household Consumption Expenditure Survey (HCES) 2010/11. | n/a; | 1.18 (US$ 1.9/ capita/ day); 1.19 (ANPL) | n/a | n/a |
| Ebaidalla & Ali, 2019 | Sudan | National Baseline Household Survey (NBHS), 2009 | n/a | 4.1 (ANPL) | n/a | n/a |
| Gabani & Guinness, 2019 | Liberia | Household Income and Expenditure Survey, 2014 | 1.8 (TE_Cata10); 0.5 (TE_Cata25); 1.8 (nFE_Cata40) | 0.6 (ANPL) | n/a | 8 |
| Edoka et al., 2017 | Sierra Leone | Integrated Household Survey, 2003 & 2011 | 32 (TE_Cata10) | n/a | n/a | n/a |
| McHenga et al., 2017 | Malawi | Integrated Household Survey (IHS), 2010/11 | 0.73 (nFE_Cata40); 9.37 (nFE_Cata10) | 0.93 (ANPL) | n/a | n/a |
| Mussa, 2016 | Malawi | Integrated Household Survey (IHS), 2010/11 | 2.43 (TE_Cata10); 0.90 (CTP_Cata40) | n/a | n/a | n/a |
| Kwesiga et al., 2015 | Uganda | National Household Survey, 2009/10 | 22.8 (TE_Cata10: variable threshold); 6.7 (TE_Cata25: variable threshold) | 4.1 (IPL US$ 1.25/ capita/day); 4.2 (ANPL) | n/a | n/a |
| **National results single-country studies: lower middle-income counties (n = 66)** | | | | | | |
| Ahmed et al., 2021 | Bangladesh | HIES 2016 | 24.6 (TE_Cata10); 10.9 (nFE_Cata40) | 4.5 (ANPL) | n/a | n/a |
| Ataguba, 2021 | Nigeria | Harmonized Nigeria National Living Standard Survey (HNLSS), 2008/09 | n/a | 0.147 (IPL US$2.2/ capita/day); 0.219 (IPL US$2.3/ capita/day); 0.163 (IPL US$2.4/ capita/day) | n/a | n/a |
| Dorjdagva et al., 2021 | Mongolia | Household Socio-Economic Survey, 2012 | 0.50 – 3.02 (CTP_Cata40) [Inpatient care at private facility – inpatient care at tertiary public hospitals, OOP includes direct medical and n/an-medical costs] | 0.09 – 0.13 (ANPL) [Inpatient care at private facility – inpatient care at tertiary public hospitals, OOP includes direct medical and n/an-medical costs] | n/a | n/a |
| Dwivedi et al., 2021 | India | National Sample Survey (NSS)1993/94, 2004/05, & 11/2012 | 17.32 (TE_Cata10); 4.04 (CTP_Cata40) | n/a | n/a | n/a |
| Iamshchikova et al., 2021 | Kyzgyz Republic | Integrated Household Surveys, 2012 - 2018 (seven rounds) | 21 (TE_Cata10), 9 (TE_Cata25), 33 (CTP_Cata40) | n/a | n/a | n/a |
| Jithitikulchai et al., 2021 | Cambodia | Cambodian Socio-Economic Survey (CSEC), 2004, 2009, 2014, 2015, 2016, 2017 | 14.0 (TE_Cata10); 5.6 (CTP_Cata40) | n/a | n/a | n/a |
| Mohanty & Dwivedi, 2021 | India | National Sample Survey (NSS), 2004 (2 surveys), 2011/12, 2014, & 2018. | 17.44 (TE_Cata10); 9.10 (CTP_Cata40) | 3.32 (RNPL) | n/a | n/a |
| Thapa & Pandey, 2021 | Nepal | Nepal Living Standard Survey, 2012 (?) | 11.11 (nFE_Cata40) | n/a | n/a | n/a |
| Thu Thuong et al., 2021 | Vietnam | Household Living Standards Survey, 2016 | 9.89 (CTP_Cata40) | n/a | n/a | n/a |
| Abdi et al., 2020 | Iran | Household Income and Expenditure survey (HIES), 2014, 2015 | 2.1 (CTP_Cata40) | 0.5 (IPL US$ 1.9/ capita/day) | n/a | n/a |
| Akhtar et al., 2020 | India | National Sample Survey (NSS)2004, & 2014; Indian Human Development Survey, 2004/05, & 2011/12 | 67.99 - 73.21 (nFE_Cata40) [outpatient – inpatient] | n/a | n/a | n/a |
| Kazemi-Karyani et al., 2020 | Iran | Household Income and Expenditure survey (HIES), 2017 | 3.32 (CTP_Cata40) | n/a | n/a | n/a |
| Molla & Chi, 2020 | Bangladesh | HIES 2010 | 12.1 (TE_Cata10); 3.7 (TE_Cata25); 8.9 (nFE_Cata40) | 3.0 (lower ANPL); 3.2 (Upper ANPL) | n/a | n/a |
| Njagi et al., 2020a | Kenya | Kenya Household Health Expenditure and Utilization (KHHEUS), 2013 | n/a | n/a | n/a | 3.2 |
| Njagi et al., 2020b | Kenya | Kenya Household Health Expenditure and Utilization (KHHEUS), 2007, & 2013 | 6.5 (CTP_Cata40) | n/a | n/a | n/a |
| Rezaei et al., 2020 | Iran | Households Income and Expenditure Survey (HIES), 1991, 1996, 2001, 2006, 2011, & 2017 | 5.26 (CTP_Cata40) | n/a | n/a | n/a |
| Sangar et al., 2020 | India | National Sample Survey (NSS), 2014 | n/a; | n/a | Distribution across inpatient and outpatient by urban and rural:   savings/income: 88.3- 98.2; borrowings: 4.3 - 41.4; sale of assets: 1.2 - 3.4; contributions: 2.4 - 16.2 | n/a |
| Ahmadnezhad et al., 2019 | Iran | Household Income and Expenditure survey (HIES), 2013, 2016 | 2.72 (TE_Cata25); 2.06 (nFE_Cata40); 1.91 (CTP_Cata40) | n/a | n/a | n/a |
| Dugee et al., 2019 | Mongolia | Household Socio-Economic Survey, 2012 | 10.5 (TE_Cata10); 3.3 (nFE_Cata40) [OOP includes direct n/an-medical costs] | 2.2 (ANPL) | n/a | n/a |
| Ergo et al., 2019 | Myanmar | Poverty and Living Conditions Survey, 2015 | 4.4 (CTP_Cata40) | 3.2 (ANPL); 3.2 (RNPL) | borrowing: 28.3; sale of assets : 12.7 | 3.67% |
| Goyanka et al., 2019 | India | National Sample Surveys: Consumption Expenditure Survey, 2004/2005, and 2011/2012; and Health and Morbidity Survey, 2004 and 2014 | n/a | 7.53 (ANPL) | n/a | n/a |
| Joshani Kheibari et al., 2019 | Iran | Household Income and Expenditure survey (HIES), 2010 -2016 (seven waves) | 3.45 (CTP_Cata40) | 0.91 (RNPL) | n/a | n/a |
| Oudmane et al., 2019 | Morocco | National Household Consumption and Expenditure Survey (NHCES), 2013/2014. | 12.31 (TE_Cata10); 1.58 (TE_Cata25); 1.63 (CTP_Cata40) | 1.11 (ANPL) | n/a | n/a |
| Rezaei & Hajizadeh, 2019 | Iran | Households Income and Expenditure Survey (HIES), 2017. | 5.26 (CTP_Cata40) | n/a | n/a | n/a |
| Salari et al., 2019 | Kenya | Kenya Household Health Expenditure and Utilization Survey (KHHEUS), 2018 | 10.7 (TE_Cata10); 7.1 (nFE_Cata40) | 2.2 (ANPL) | n/a | n/a |
| Sangar et al., 2019a | India | National Sample Survey (NSS), 2014 | 22.9 (TE_Cata10); 11.8 (TE_Cata25) | 8.0 (ANPL) | n/a | n/a |
| Sangar et al., 2019b | India | National Sample Survey (NSS), 2014 | 23.7 (TE_Cata10) | 8.0 (PL n/at specified) | n/a | n/a |
| Sangar et al., 2019c | India | National Sample Survey (NSS), 2004, & 2014 | n/a; | n/a | borrowing: 6.6 - 37.6; other sources: 3.7 - 19.3 [outpatient - inpatient] | n/a |
| Sangar et al., 2019d | India | National Sample Survey (NSS), 2014 | 23.7 (TE_Cata10); 12.4 (TE_Cata25); 40.5 (TE_Cata_base) | 8.0 (ANPL) | Savings: 88.9 - 97.8 ; borrowing: 6.6 - 37.3 ; sale of assets: 0.05 - 1.2, contribution from family and friends: 3.5 - 15.3; other sources: 1.2 - 2.1 [outpatient- inpatient] | n/a |
| Yazdi-Feyzabadi et al., 2019 | Iran | Households Income and Expenditure Survey (HIES), 2011 - 2017 (seven waves) | 3.46 (CTP_Cata40) | n/a | n/a | n/a |
| Zhang et al., 2019 | Ghana | Ghana Living Standard Survey, 1991/92, 1998/99, 2005/06, & 2012/13 | 3.2 (nFE_Cata25) | 0.6 (RNPL) | n/a | n/a |
| Ahmed et al., 2018 | Vietnam | Household Living Standards Survey, 2012 | 11.7 -19.0 (TE_Cata10); 24.0 - 30.2 (CTP_Cata40) [Mekong Delta region and the rest of Vietnam] | 2.3 – 5.0 (ANPL) [Mekong Delta region and the rest of Vietnam] | n/a | n/a |
| Antunes et al., 2018 | Cambodia | Cambodian Socio-Economic Survey (CSEC), 2004, 2009, 2014 | 4.9 (CTP_Cata40) | 1.4 (RNPL) | n/a | n/a |
| Aregbeshola & Khan, 2018 | Nigeria | Harmonized Nigeria Living Standard Survey (HNLSS), 2009/2010 | 16.4 (TE_Cata10); 13.6 (TE_Cata25); 13.7 (nFE_Cata40) | 0.8 (IPL US$1.25 / capita/day); 0.4 (IPL US$ 2.0/ capita/day) | n/a | n/a |
| Arenliu Qosaj et al., 2018 | Kosovo | Household Budget Survey (HBS), 2014 | 13.01 (TE_Cata10); 1.69 (TE_Cata25); 5.34 (nFE_Cata40) | 1.5 (ANPL) | n/a | n/a |
| Barasa et al., 2018 | Kenya | Kenya Household Expenditure and Utilization Survey (KHEUS), 2003, 2007, & 2013 | 4.52 (CTP_Cata40) | 39.14 (ANPL) [Including further impoverished = 38.12] | n/a | n/a |
| Ghiasvand et al., 2018 | Iran | Household Income and Expenditure survey (HIES), 2002/03 - 2013/14 (11 waves) | 0.48 - 0.52 (CTP_Cata40) (urban - rural) | 0.03 (RNPL) [both urban and rural] | n/a | n/a |
| Ghimire et al., 2018 | Nepal | Living Standards Survey, 2010/11 | 10.3 (CTP_Cata40) | n/a | n/a | n/a |
| Han et al., 2018 | Myanmar | Integrated Household Living Condition Assessment, 2009/10 | 14.6 (nFE_Cata40) | 2.0 (ANPL) | n/a | n/a |
| Mohanty et al., 2018 | India | National Sample Survey (NSS)2011/12 | 23.4 (CTP_Cata40) | n/a | n/a | n/a |
| Moradi et al., 2018 | Iran | Households Income and Expenditure Survey (HIES), 2015 & 2016 | 2.5 – 3.6 (CTP_Cata40) (urban – rural) | n/a | n/a | n/a |
| Ngcamphalala & Ataguba, 2018 | Swaziland (n/aw Eswatini) | Household Income and Expenditure Survey, 2009/2010 | 9.7 (TE_Cata10: variable threshold); 2.4 (TE_Cata25: variable threshold) 2.7 (nFE_Cata40: variable threshold) | 1.6 (IPL US$ 1.25/ capita/ day:); 1.0 (ANPL) | n/a | n/a |
| Pandey et al., 2018 | India | National Sample Survey (NSS)2004, & 2014 | 21.2 (TE_Cata10) | n/a | n/a | n/a |
| Pandey, Ploubidis, et al., 2018 | India | National Sample Survey (NSS), 1993/94, 1999/2000, 2004/05, 2011/12, 1995/96, 2004, & 2014) | 24.9 (TE_Cata10) | n/a | n/a | n/a |
| Rahman et al., 2018 | Bangladesh | HES, 1991/92, 1995/96; HIES 2000, 2005, 2010,  2005, 2010; UHES, 2011; BIHS, 2011/12 | 16.4 (TE_Cata10); 9.7 (nFE_Cata40); 3.4 (CTP_Cata40) | 2.8 (RNPL) | n/a | n/a |
| Sangar et al., 2018 | India | National Sample Survey (NSS), 2004, & 2014 | 24.8 - 28.6 (TE_Cata10) [rural – urban] | 7.9 - 8.4 (ANPL) [rural – urban] | borrowing: 4.1 – 28.9; others: 13.3 – 14.2 [outpatient-inpatient by rural-urban areas] | n/a |
| Selvaraj et al., 2018 | India | National Sample Survey (NSS), 1993/94, 2004/05, 2011/12, 2014 | 17.9 (TE_Cata10); 4.3 (TE_Cata25); 4.9 (nFE_Cata40) | 4.04 (IPL US$ 1.9/capita/day); 4.48 (ANPL) | n/a | n/a |
| Swe et al., 2018 | Nepal | Living Standards Survey, 1995, & 2010 | 15.3 (TE_Cata10); 12.2 (nFE_Cata40); 9.6 (CTP_Cata40) | 3.0 (ANPL) | n/a | n/a |
| Yazdi-Feyzabadi et al., 2018 | Iran | Households Income and Expenditure Survey (HIES), 2008 - 2015 (eight waves) | 3.25 (CTP_Cata40) | n/a | n/a | n/a |
| Zeng et al., 2018 | Zimbabwe | National household survey, 2016 | 12.63 (TE_Cata10); 7.64 (TE_Cata25) | 1.29 (ANPL) | n/a | n/a |
| Ahmad & Aggarwal, 2017 | India | Indian Human Development Survey (IHDS-II), 2012 | 13.21 -15.4 (TE_Cata10); 4.42 - 5.17 (TE_Cata25); 3.68 -4.49 (nFE_Cata30) [HHs engaged in formal sector - HHs engaged in informal sector] | 2.56 - 7.12 (ANPL) [HHs engaged in formal sector - HHs engaged in informal sector] | n/a | n/a |
| Aji et al., 2017 | Indonesia | Indonesian Family Life Surveys 1993, 1997, 2000, & 2007 | 4.33 (TE_Cata10); 1.14 (TE_Cata25) | 0.63 (IPL US$ 1.08/ capita/day); 1.53 (IPL US$2.15 / capita/day) | n/a | n/a |
| Akazili et al., 2017 | Ghana | Ghana Living Standard Survey, 2005/06 | n/a; | 9.4 (IPL US$1.25 / capita/day); 3.8 (IPL US$ 2.50/ capita/day) | n/a | n/a |
| Akazili, McIntyre, et al., 2017 | Ghana | Ghana Living Standard Survey, 2005/06 | 2.24 (TE_Cata10); 2.64 (nFE_Cata40) | n/a | n/a | n/a |
| Ayadi & Zouari, 2017 | Tunisia | National Budget and Consumption Survey, 2000, 2005, & 2010 | 16.11 (TE_Cata10); 7.01 (nFE_Cata25) | 1.49 (RNPL) | n/a | n/a |
| Barasa et al., 2017 | Kenya | Kenya Household Expenditure and Utilization Survey (KHEUS), 2003, and 2013 | 4.52 (nFE_Cata40) | 1.17 (ANPL) | n/a | n/a |
| Khan et al., 2017 | Bangladesh | HIES, 2010 | 14.2 (TE_Cata10); 9.7 (nFE_Cata40) | 3.5 (ANPL) | n/a | n/a |
| Amaya-Lara, 2016 | Colombia | Quality of Life National Survey, 2011 | 9.6 (CTP_Cata20) | n/a | n/a | n/a |
| Amponsah, 2016 | Ghana | Ghana Living Standard Survey, 1998/99, 2005/06, & 2012/13 | 5.47 (nFE_Cata10) | n/a | saving: 15.7; borrowing from others: 8.1; contribution: 2.1 |  |
| Bredenkamp & Buisman, 2016 | Philippines | Family Income and Expenditure Surveys (FIES), 2000, 2003, 2006, 2009, & 2012 | 7.7 (TE_Cata10); 1.9 (TE_Cata25); 2.3 (nFE_Cata40) | 1.0 (IPL US$1.25 / capita/day); 1.5 (IPL US$2.00 / capita/day) (2012) | n/a | n/a |
| Cros et al., 2019 | Haiti | Household Living Conditions survey, (Enquete sur les Conditions de Vie des Menages Apres Seisme, ECVMAS), 2012 & 2013 | 11.54 (TE_Cata10) | n/a | n/a | 13.40% |
| Dorjdagva et al., 2016 | Mongolia | Household Socio-Economic Survey, 2012 | 5.0 (TE_Cata10); 0.9 (TE_Cata25); 0.8 (nFE_Cata40) | 0.03 (IPL US$1.9/ capita/ day); | n/a | n/a |
| Jacobs et al., 2016 | Cambodia | Cambodian Socio-Economic Survey (CSEC), 2004, 2009, 2014 | 4.3 – 6.5 (CTP_Cata40) [households without older people -households with older people] | 0.9 - 1.0 (ANPL) [households without older people -households with older people] | having at least one active loan whose main purpose was to pay for the care of an illness, injury or accident: 2.2 - 2.5 (households with older people -households with older people) | n/a |
| Masiye et al., 2016 | Zambia | Household Health Expenditure and Utilisation Survey, 2014 | 4.2 (TE_Cata10); 4.0 (CTP_Cata40) [outpatient care from formal providers] | n/a | n/a | n/a |
| Htet, Fan, et al., 2015 | Myanmar | World Health Survey (WHS), 2002/03 | 41 (CTP_Cata40) (2002/03) | n/a | borrowing or sale of assets: 5 | n/a |
| Séne & Cissé, 2015 | Senegal | Poverty Monitoring Survey, 2011 | 6.77 (TI_Cata10); 1.10 (TE_Cata25) | 1.44 (ANPL) (among HHs who face CHE at the 10% of total income threshold) | n/a | n/a |
| **National results single-country studies: upper middle-income counties (n = 12)** | | | | | | |
| Liu et al., 2021 | China | China Family Panel Studies (CFPS) survey, 2010, 2012, 2014, 2016, & 2018 | 8.7 (CTP_Cata40) | n/a | n/a | n/a |
| Taniguchi et al., 2021 | Iraq | Household Socio-Economic Survey (HSES), 2006/07, & 2012 | 12.4 (TE_Cata10) | 2.8 (PL not specified) | n/a | n/a |
| Sun & Lyu, 2020 | China | China Family Panel Studies (CFPS), 2012, 2014, & 2016. | 15.24 (nFE_Cata40) | n/a | n/a | n/a |
| Wang et al., 2020 | China | National Health Service Survey (NHSS), 2013 | 14.43% - 15.53% (CTP_Cata40) [different insurance schemes] | 6.07 - 6.41 (RNPL) [different insurance schemes] | n/a | n/a |
| Hernández-Vásquez et al., 2020 | Peru | National Household Survey on Living Conditions (Encuesta Nacional de Hogares, ENAHO), 2008, and 2017 | 3.7 (nFE_Cata40) | n/a | n/a | n/a |
| Tangcharoensathien et al., 2020 | Thailand | National Household Socio-Eeconomic surveys (SES), 1996, 1998, 2000, 2002, 2004, 2006, 2007, 2008, 2009, 2010, 2011, 2012, 20013, 2014, & 2015 | 2.0 (TE_Cata10); 0.4 (TE_Cata25) | 0.07 (IPL US$ 1.9/capita/day); 0.4 (IPL US$ 3.1/capita/day); 0.3 (ANPL) | n/a | n/a |
| Manavgat et al., 2020 | Turkey | Household Budget Surveys, 2002-2016 (eight waves) | 0.33 (CTP_Cata40); 1.38 (CTP_Cata40: variable threshold) | n/a | n/a | n/a |
| Ma et al., 2019 | China | China Family Panel Studies (CFPS) survey, 2010, 2012, 2014, & 2016 | 25.09 (TE_Cata10); 9.96 (TE_Cata25); 8.94 (CTP_Cata40) | n/a | n/a | n/a |
| Nikoloski & Mossialos, 2018 | Mexico | Survey of Health and Nutrition (Encuesta Nacional de Salud y Nutricion, ENSANUT), 2006, 2012 | 2.6 – 2.9 (TE_Cata30) [insured – uninsured] | 0.87 (IPL US$1.9/capita/day); 1.63 (IPL US$ 3.1/capita/day) | n/a | n/a |
| Falconi & Bernabe, 2018 | Peru | National Household Survey (Encuesta Nacional de Hogares, ENAHO), 2016 | 4.09 (CTP_Cata40) | n/a | n/a | n/a |
| Abeldaño, 2017 | Argentina | National Survey of Household Expenditure 2012/13 | 2.3 (TE_Cata30) | 1.7 (ANPL) | n/a | n/a |
| Narci et al., 2015 | Turkey | Household Budget Surveys, 2004 - 2010 (seven waves) | 0.75 (CTP_Cata40) | 0.01 (IPL US$ 1.0/ capita/day); 0.09 (IPL US$2.15/ capita/ day); 0.58 (US$4.3/ capita/ day); 0.25 (ANPL, food); 1.18 (ANPL, food and n/an-food)); 1.00 (RNPL) | n/a | n/a |
| **Subgroup results: multi-country studies (n = 1)** | | | | | | |
| Kumar et al., 2015 | multicountry (China & India) | WHO Study on lobal AGEing and adult health (SAGE); 2007/2010 | n/a | 7.4 - 8.0 (RNPL) | n/a | n/a |
| **Subgroup results single-country studies: lower-middle-income countries (n = 4)** | | | | | | |
| Kastor & Mohanty, 2018 | India | National Sample Survey (NSS), 2014 | 49.0 (TE_Cata10) [inpatient care] | n/a | borrowing, sale of assets, contributions from friends and relative: 28.6; highest for cancer treatment: 42.5 (2014), lowest for cataract: 15.7 (2014), | n/a |
| Lee et al., 2018 | India | National Sample Survey (NSS), 2014 | 33 - 39 (TE_Cata10); | 4 - 5 (IPL US$ 1.90/capita/ day) | n/a | n/a |
| Ranjan et al., 2018 | India | National Sample Survey (NSS), 2014 | 39.62 (TE_Cata10); 18.22 (TE_Cata25) | 12.73 (ANPL) | n/a | n/a |
| Brinda et al., 2015 | India | WHO's Study on global AGEing and adult health India (SAGE), 2007/08 | 7 (CTP_Cata40) | n/a | n/a | n/a |
| **Subgroup results single-country studies: upper-middle-income countries (n = 10)** | | | | | | |
| Fu, 2021 | China | China Household Finance Survey (CHFS), 2015, 2017 | 14.68 (nFE_Cata40) | 5.32 (IPL US$ 1.9/ capita/day) | n/a | n/a |
| Zhang, Dong, et al., 2021 | China | China National Health Services Survey (NHSS), 2003, 2008, and 2013. | 8.7 (TI_Cata40) [incidence among households enrolled in a particular insurance scheme] | n/a | n/a | Individual-level analysis: Ill individual did not get admitted when considered necessary by physicians: 21.4%; Ill individual forwent necessary admissions due to financial difficulties: 45.4%; ill individual took early discharge due to financial difficulties: 37.0% |
| Zhou et al., 2021 | China | China Health and Retirement Longitudinal Study (CHARLS), 2011, 2013, & 2015 | 27.9 (nFE_Cata40) | n/a | n/a | n/a |
| Ma et al., 2020 | China | China Health and Retirement Longitudinal Study (CHARLS), 2015 | 20.3 (CTP_Cata40) [ for households with middle-aged and elderly population aged 45 years and over] | 7.2 (RNPL) | n/a | n/a |
| Xu et al., 2020 | China | China Health and Retirement Longitudinal Study (CHARLS), 2013 | 23.46 (CTP_Cata40) | n/a | n/a | n/a |
| Yang, 2020 | China | China Health and Retirement Longitudinal Survey (CHARLS), 2015 | 2.63 (nFE_Cata40) [Inpatient care] | 0.98 (US$ 1.9/capita/day) [Inpatient care]; 0.71 (ANPL) [Inpatient care] | n/a | n/a |
| Zhong et al., 2020 | China | China Health and Retirement Longitudinal Study (CHARLS), 2011, & 2013 | 15.56 (nFE_Cata40) | n/a | n/a | n/a |
| Meemon & Paek, 2019 | Thailand | Health and Welfare Survey (HWS) 2015 | 2.78 [inpatient services] | n/a | n/a | n/a |
| Li et al., 2017 | Jamaica | Jamaica Survey of Living Conditions (JSLC), 1996 - 2012 (14 waves excluding 2003, 2005, & 2011 rounds) | 0.6 (nFE_Cata40) [among households with under-18 children who fell ill in the past 4 weeks before the survey] | n/a | n/a | n/a |
| Doubova et al., 2015 | Mexico | Survey of Health and Nutrition (Encuesta Nacional de Salud y Nutricion, ENSANUT), 2012 | 2.02 - 3.9 (CTP_Cata40); [elderly HHs with insurance – elderly HHs without insurance] | n/a | 14.8 - 20.0 [elderly HHs with insurance – elderly HHs without insurance] | 10.2 - 57.8 [insured elderly people– uninsured elderly people] |

Note: Incidences of financial protection indicators are for the latest year of data analyzed in each study

HH = Households

CHE = Catastrophic health expenditure,

TE_CataX = CHE measured through the budget-share method; Denominator: total expenditure, Threshold: X%

TI_CataX = CHE measured through the budget-share method; Denominator: total income, Threshold: X%

nFE_CataX = CHE measured through the actual food expenditure method; Denominator: total non-food expenditure, Threshold: X%

CTP_CataX = CHE measured through the capacity-to-pay or the normative food expenditure method; Denominator: total non-subsistence expenditure, Threshold: X%

PL = Poverty line, IPL = International poverty line, ANPL = Absolute national poverty line, RNPL = Relative national poverty line,

pp = percentage points
