# Supplementary material for: Financial risk protection from out-of-pocket health spending in low- and middle-income countries: a scoping review of the literature
Source: Health Res Policy Syst. 2022 Jul 29;20:83. doi: 10.1186/s12961-022-00886-3 (PMC9336110; doi:10.1186/s12961-022-00886-3)
Supplement: Supplementary file 4 — Additional file 4. Financial risk protection against chronic diseases including noncommunicable diseases and injuries. The studies on financial risk protection against chronic diseases including noncommunicable diseases and injuries are summarized by author(s) name and year, country, data source, disease (study subgroup), incidences of catastrophic health expenditure, impoverishment, and coping. [file 12961_2022_886_MOESM4_ESM.docx]

**Additional file 4**: Financial risk protection against chronic diseases including non-communicable diseases and injuries

| **Study** | **Country** | **Data Source** | **Disease (study subgroup)** | **Incidence of CHE (%)** | **Incidence of impoverishment (percentage point)** | **Incidence of Coping (%)** |
| --- | --- | --- | --- | --- | --- | --- |
| **National results: multi-country studies (n = 1)** |  |  |  |  |  |  |
| Alam & Mahal, 2016 | 5 LwMICs in South Asian countries | World Health Survey data, 2002/03 | injury | 33.81 (CTP_Cata40) | n/a | borrowing or sale of assets: 38.46 |
| **National results: lower middle-income countries (n = 12)** |  |  |  |  |  |  |
| Ahmed et al., 2021 | Bangladesh | Household Income and Expenditure Survey (HIES), 2016 | chronic illnesses | 39.7 (TE_Cata10); 17.8 (nFE_Cata40) | n/a | n/a |
| Kazemi-Karyani et al., 2020 | Iran | Household Income and Expenditure Survey (HIES), 2017 | special chronic diseases (e. g., thalassemia, cancer) | 19.29 (CTP_Cata40) | n/a | n/a |
| Njagi et al., 2020b | Kenya | Household Health Expenditure and Utilisation (KHHEUS), 2007 and 2013. | chronic illnesses | 3.2 (CTP_Cata40) | n/a | n/a |
| Datta et al., 2019 | Pakistan | Household Integrated Economic Survey, 2015/16 | hypertension and diabetes | 12.96 (TE_Cata10) | n/a | n/a |
| Dugee et al., 2019 | Mongolia | Household Socioeconomic Survey, 2012 | chronic diseases | 10.5* (TE_Cata10); 3.3* (nFE_Cata40) | 7.9 (ANPL) | n/a |
| Sangar et al., 2019b | India | National Sample Survey, 2014 | NCDs | NCDs: 15.8, Injuries: 1.3 (TE_Cata10) | (**PL not specified**):  NCDs: 5.4  Injuries: 0.5 | n/a |
| Sepehri & Vu, 2019 | Vietnam | Vietnam Household Living Standard Survey of 2014 | severe injury (urban -rural) | (urban - rural): 24.3 -32.8 (CTP_Cata20) | n/a | n/a |
| Datta et al., 2018 | Bangladesh | HIES 2010 | six major NCDs | 9.5 (TE_Cata10) | 1.81 (ANPL) | borrowing or sale of assets: 7.82 |
| Ghimire et al., 2018 | Nepal | Nepal Living Standards Survey, 2010/11 | chronic illnesses | 1.5 (CTP_Cata40) | n/a | n/a |
| Swe et al., 2018 | Nepal | Nepal Living Standards Survey, 1995 and 2010. | chronic diseases and injuries | **chronic diseases:** 9.6 (TE_Cata10), 4.1 (nFE_Cata40), 3.4 (CTP_Cata40); I**njuries:** 22.25 (TE_Cata10), 22.25 (TE_Cata10), 15.0 (nFE_Cata40), 14.2 (CTP_Cata40) | chronic diseases: 1.3 (ANPL); Injuries: 2.2 (ANPL); | n/a |
| Tripathy & Prasad, 2018 | India | National Sample Survey, 2014 | diabetes (outpatient - inpatient) | (outpatient- inpatient): 0 - 17 (TE_Cata30) | n/a | borrowings/sale of physical assets: (outpatient - inpatient) 1 - 20 |
| Tripathy et al., 2018 | India | National Sample Survey, 2014 | injury (outpatient - inpatient) | (outpatient - inpatient): 2 - 25 (TE_Cata30) | n/a | borrowings/sale of assets: 8% (outpatient) - 27% (inpatient) (2014) |
| **National results: upper middle-income countries (n = 2)** |  |  |  |  |  |  |
| Hernández-Vásquez et al., 2020 | Peru | National Household Surveys on Living and Poverty Conditions (Encuesta Nacional de Hogares, ENAHO in Spanish), 2008 and 2017 | chronic diseases | 4.5 (nFE_Cata40) | n/a | n/a |
| Falconi & Bernabe, 2018 | Peru | Peru National Household Survey (Encuesta Nacional de Hogares, ENAHO in Spanish), 2016 | chronic illnesses (one individual with chronic disease - three or more individuals with chronic disease) | 3.93 - 5.01 (CTP_Cata40) | n/a | n/a |
| **Subgroup results: multi-country studies (n= 2)** |  |  |  |  |  |  |
| Gwatidzo & Williams, 2017 | China and India | World Health Organization (WHO) Study on global AGEing and adult health (SAGE) Wave 1 (2007-2010) | diabetes (among people aged 50 years and over) | 6.6 - 16.8 (TI_Cata40) | n/a | n/a |
| Goeppel et al., 2016 | six countries (2 LwMIC, and 4 UMIC) | World Health Organization Study on Global Ageing and Adult Health, 2007-2010 | chronic illnesses (among people aged 50 years and over) | 23.5 - 65.5 (TI_Cata30) | n/a | n/a |
| **Subgroup results: lower middle-income countries (n = 9)** |  |  |  |  |  |  |
| Marthias et al., 2021 | Indonesia | Indonesian Family Life Survey, 2007 and 2014 | NCDs (among people aged 50 years and above with one NCD to three or more NCDs) | 6.9 - 12.5 (TE_Cata10); 1.5 - 2.8 (TE_Cata25); 2.7 - 3.6 (CTP_Cata40) | n/a | n/a |
| Rajasulochana & Kar, 2021 | India | National Sample Survey 2017/18 | stroke (inpatient care) | 58 (TE_Cata10) | n/a | borrowing or sale of assets for inpatient care for stroke: 37% (2017/18), |
| Verma et al., 2021 | India | National Sample Survey, 2014 and 2017/18 | NCDs (among HHs seeking NCD care) | 63.61* (TE_Cata10); 39.62* (TE_Cata25) | 12.43 (ANPL) | no |
| Yadav et al., 2021 | India | National Sample Survey, 2004, 2014, and 2018 | NCDs (inpatient care at public and private facility) | **any NCD:** 47.2 (TE_Cata10)**;** [Major NCDs: Cancers: 70.3, stroke: 63.5, cardiovascular diseases (CVD): 50.5] | **any NCD:** 4.8 (IPL US$ 1.9/ capita/ day)**; [Major NCDs:** Cancers: 12.3, cardiovascular diseases: 6.9, stroke: 5.6] | borrowing with interest or sale of assets: any NCDs: 12.7% (2018) |
| Yadav, Menon, et al., 2021 | India | National Sample Survey, 2017/18 | injury (inpatient care among 15 - 59 years old population) | Injury: 58.8 (TE_Cata10) | n/a | n/a |
| Ranjan et al., 2019 | India | National Sample Survey, 2014 | rheumatic disease (inpatient care) | 53.8 (TE_Cata10) | 15.6 (ANPL) | borrowing or sale of assets: 24.3, |
| Kastor & Mohanty, 2018 | India | 71st round of the National Sample Survey Organization (2014) | NCDs and injuries (inpatient care) | NCDs: 58 (TE_Cata10); Injuries: 52 (TE_Cata10) | n/a | borrowing, sale of assets, contributions from friends and relative: 33.0% (2014) |
| Rajpal et al., 2018 | India | National Sample Survey, 2014 | cancer (inpatient care at public and private facility) | (Public facility - private facility): 36.3 - 63.8 (TE_Cata10), | n/a | borrowings, sale of assets, and contribution: > 40% (public facility) - 50% (private facility) (2014) |
| Tripathy et al., 2016 | India | National Sample Survey, 2014 | NCDs including injuries (inpatient care) | Overall NCDs including injuries: 53 (TE_Cata10); [Major NCDs: cancer: 74,  CVDs: 53; and Injuries: 55] | n/a | borrowings/ sale of assets: overall NCDs: 26, [Major NCDs: Cancer: 36, Cardiovascular:24, and Injuries: 28] |
| **Subgroup results: upper middle-income countries (n = 12)** |  |  |  |  |  |  |
| Ma et al., 2021 | China | China Health and Retirement Longitudinal Study (CHARLS), 2015 | cardiovascular diseases (among people aged 45 years and above) | 7.6 (TE_Cata10); 19.9 (CTP_Cata40) | 7.6 (RNPL) | n/a |
| Wang et al., 2021 | China | China Health and Retirement Longitudinal Study (CHARLS), 2011, 2013, and 2015 | NCDs (among people aged 45 years and over) | 24 (CTP_Cata40) | 8.8 (RNPL) | n/a |
| X. Z. Fu et al., 2021 | China | China Family Panel Studies, 2012, 2014, 2016, and 2018 | NCDs (among HHs with members suffering from NCDs in urban and rural areas) | (urban - rural): 17.96 - 26.14 (nFE_Cata40) | n/a | n/a |
| Zhang, Dong, et al., 2021 | China | China National Health Services Survey, 2003, 2008, and 2013. | NCDs (people enrolled in a particular insurance scheme) | 14.8 (TI_Cata40) | n/a | n/a |
| Bernardes et al., 2020 | Brazil | Estudo Longitudinal de Saude dos Idosos Brasileiros (ELSI - Brazilian Longitudinal Study of Aging), 2015/16 | Chronic illnesses (among people aged 50 years and over) | 17.9 (TE_Cata10); 7.5 (TE_Cata25) | n/a | n/a |
| Y. Zhao et al., 2020 | China | China Health and Retirement Longitudinal Study (CHARLS), 2011, 2013, and 2015 | NCDs (among people aged 45 years and over with one NCD to four or more NCDs); [incidence from pooled panel data from 3 rounds: 2011, 2013, 2015] | 16.2 - 30.8 (nFE_Cata40) | n/a | n/a |
| Y. Zhao, B. Oldenburg, et al., 2020 | China | China Family Panel Studies 2010, 2012, 2014, and 2016 | NCDs (among adults aged 16 years and over with one chronic disease to two or more chronic diseases) | 23.08 - 30.58 (nFE_Cata40) | (one chronic disease - two or more chronic diseases): 7.83 - 11.72 (IPL US$ 1.9/ capita/day) | n/a |
| Y. Zhao, L. Zhang, et al., 2020 | China | China Health and Retirement Longitudinal Study (CHARLS) , 2015 | Cancer (among 45 years and older) | 26.77 (nFE_Cata40) | n/a | n/a |
| Yang, 2020 | China | China Health and Retirement Longitudinal Study (CHARLS) , 2015 | NCDs (inpatient care among people aged 60 and over) | 2.59* (nFE_Cata40) | 1.02 (IPL US$ 1.9/capita/day); 0.70 (ANPL) | n/a |
| Meemon & Paek, 2019 | Thailand | Health and Welfare Survey (HWS) 2015 | chronic diseases (inpatient care sought by non-single-person HHs and single-person HHs) | 2.60 - 6.76 (CTP_Cata40) | n/a | n/a |
| Li et al., 2018 | China | China Health and Retirement Longitudinal Study (CHARLS), 2011 and 2013 | cardiovascular diseases (among people aged 45 years and above) | 48.1 (nFE_Cata40) | n/a | n/a |
| Wang et al., 2015 | China | China Health and Retirement Longitudinal Study (CHARLS), 2011 | NCDs (among people aged 45 years and over in urban and rural areas) | 22.03 - 30.57 (nFE_Cata40) | n/a | n/a |

Note: Incidences of financial protection indicators are for the latest year of data analyzed in each study.

LwMIC = Lower middle-income country

NCDs: Non-communicable diseases

HH = Households

CHE = Catastrophic health expenditure,

TE_CataX = CHE measured through the budget-share method; Denominator: total expenditure, Threshold: X%

TI_CataX = CHE measured through the budget-share method; Denominator: total income, Threshold: X%

nFE_CataX = CHE measured through the actual food expenditure method; Denominator: total non-food expenditure, Threshold: X%

CTP_CataX = CHE measured through the capacity-to-pay or the normative food expenditure method; Denominator: total non-subsistence expenditure, Threshold: X%

PL = Poverty line, IPL = International poverty line, ANPL = Absolute national poverty line, RNPL = Relative national poverty line
