# Supplementary material for: Financial risk protection from out-of-pocket health spending in low- and middle-income countries: a scoping review of the literature
Source: Health Res Policy Syst. 2022 Jul 29;20:83. doi: 10.1186/s12961-022-00886-3 (PMC9336110; doi:10.1186/s12961-022-00886-3)
Supplement: Supplementary file 5 — Additional file 5. Financial risk protection against communicable and infectious diseases. The studies on financial risk protection against communicable and infectious diseases are summarized by author(s) name and year, country, data source, disease(s)/condition(s), incidences of catastrophic health expenditure, impoverishment, and coping. [file 12961_2022_886_MOESM5_ESM.docx]

**Additional file 5:** Financial risk protection against communicable and infectious diseases

| **Study** | **Country** | **Data Source** | **Disease(s)/ Condition(s)** | **Incidence of CHE (%)** | **Incidence of Impoverishment**  **(% point)** | **Incidence of Coping (%)** |
| --- | --- | --- | --- | --- | --- | --- |
| **Low-income country (n = 1)** | | | | | | |
| Assebe et al., 2020 | Ethiopia | Nationwide household survey for HIV, 2016 | HIV | 20 (TE_Cata10); 11 (nFE_Cata40) | n/a | HIV: any coping strategy: 24%; Borrowing: 9%, sale of assets: 2%, contribution from family and friends: 12% |
| **Lower middle-income country (n = 12)** | | | | | | |
| Chittamany et al., 2020 | Lao PDR | National TB Prevalence Survey, 2010/11 | TB only and TB-HIV | all TB: 62.6**; DS-TB: 62.2**, DR-TB: 86.7**, HIV-TB: 81.1** (TI_Cata20) | n/a | saving or borrowings or sale of assets: all TB: 49.9%; DS-TB: 49.9%; DR-TB: 50.0%; TB-HIV: 65.0% |
| Dugee et al., 2019 | Mongolia | Household Socioeconomic Survey, 2012 | infectious diseases | 10.5* (TE_Cata10); 3.3* (nFE_Cata40) | 4.4*(ANPL) | n/a |
| Kastor & Mohanty, 2018 | India | National Sample Survey (NSS), 2014 | inpatient care for communicable diseases | 35 (TE_Cata10) | n/a | borrowing, sale of assets, contributions from family and friends: 23.5% |
| Negin et al., 2017 | South Africa | Study on global AGEing and adult health (SAGE) South Africa Wave 1, 2007/08 | HIV | 8.7 (CTP_Cata40) | n/a | n/a |
| Nhung et al., 2018 | Vietnam | A national survey on TB patients, 2016 | TB | all TB: 15.0, DS-TB: 9.5, MDR-TB: 77.0 (TE_Cata10); all TB: 63.0**, DS-TB: 59.6**, MDR-TB: 98.0** (TE_Cata10) | n/a | borrowing or dissaving or sale of assets: 38%; saving: 16%, borrowing: 25%; sale of assets: 5.8% |
| Pedrazzoli et al., 2018 | Ghana | A nationally representative survey on TB patients, 2016 | TB (patients availing free treatment at public health facilities) | all TB: 64.1**; DS-TB: 63.2**; MDR-TB: 72.7** (TI_Cata20) | all TB: 14.2** (IPL US$ 1.90/capita/day) | borrowing or dissaving or sale of assets: all TB: 51.5%; DS-TB: 52.0%; MDR-TB: 47.0% |
| Pedrazzoli et al., 2021 | Ghana | First national TB patient cost survey, 2016 | TB (patients availing free treatment at public health facilities) | 49** (TE_Cata10); 65** (TI_Cata20) | n/a | n/a |
| Prasad et al., 2021 | India | National Sample Survey (NSS), 1995/96, 2004/05, 2014/15, and 2017/18 | TB [outpatient - outpatient] | 0.0* -18.0* (TE_Cata20) | n/a | n/a |
| Sangar et al., 2019b | India | National Sample Survey, 2014 | infectious diseases | 6.6 (TE_Cata10) | 2.1 (PL not specified) | n/a |
| Timire et al., 2021 | Zimbabwe | Nationally representative health facility-based survey on TB patients, 2018 | TB | all TB: 80**, DS-TB: 79**, DR-TB: 90** (CTP_Cata20) | n/a | n/a |
| Tripathy et al., 2016 | India | National Sample Survey (NSS), 2014 | inpatient care for communicable diseases | 28 (TE_Cata10) | n/a | n/a |
| Yadav, John, et al., 2021 | India | National Sample Survey (NSS), 1995/96, 2004/05, 2013/14, and 2017/18 | TB | 49.3* - 52.2* (TE_Cata10) [inpatient - outpatient] | 14.2* -23.2* (ANPL) [outpatient - inpatient] | borrowing with interest and sale of assets: 5.3 - 18.3% (outpatient - inpatient) |

Note: Incidences of financial protection indicators are for the latest year of data analyzed in each study.

*OOP = direct medical costs + direct non-medical costs (such as transportation, food, lodging etc.) besides direct medical costs

** = OOP includes direct (medical and non-medical) costs and indirect costs (such as lost income)

CDs = Communicable diseases

HH = Households

HIV = Human Immunodeficiency Virus

TB = Tuberculosis

CHE = Catastrophic health expenditure,

TE_CataX = CHE measured through the budget-share method; Denominator: total expenditure, Threshold: X%

TI_CataX = CHE measured through the budget-share method; Denominator: total income, Threshold: X%

nFE_CataX = CHE measured through the actual food expenditure method; Denominator: total non-food expenditure, Threshold: X%

CTP_CataX = CHE measured through the capacity-to-pay or the normative food expenditure method; Denominator: total non-subsistence expenditure, Threshold: X%

PL = Poverty line, IPL = International poverty line, ANPL = Absolute national poverty line, RNPL = Relative national poverty line,
