# Supplementary material for: Financial risk protection from out-of-pocket health spending in low- and middle-income countries: a scoping review of the literature
Source: Health Res Policy Syst. 2022 Jul 29;20:83. doi: 10.1186/s12961-022-00886-3 (PMC9336110; doi:10.1186/s12961-022-00886-3)
Supplement: Supplementary file 6 — Additional file 6. Financial risk protection against other conditions. The studies on financial risk protection against other conditions are summarized by author(s) name and year, country, data source, conditions, incidences of catastrophic health expenditure, impoverishment, and coping. [file 12961_2022_886_MOESM6_ESM.docx]

**Additional file 6:** Financial risk protection against maternal and perinatal conditions

| **Study** | **Country** | **Data Source** | **Conditions** | **CHE (%)** | **Impoverishment (percentage point)** | **Incidence of Coping (%)** |
| --- | --- | --- | --- | --- | --- | --- |
| **Lower middle-income countries (n=4)** | | | | | | |
| Goli et al., 2016 | India | National Sample Survey, 2014 | maternity care (antenatal, delivery, postnatal) | 51.39* (TE_Cata10) | n/a | n/a |
| Mohanty & Kastor, 2017 | India | National Sample Survey, 2014 | maternity care (antenatal, delivery, postnatal); and delivery care in public and private health facilities | maternity: 29.4 - 47.2; delivery: 20.5 - 34.2 (CTP_Cata40) | n/a | n/a |
| Singh et al., 2016 | India | National Sample Survey, 2014 | maternity care (antenatal, delivery, postnatal) | 26.94 (TE_Cata10); 63.1 (CTP_Cata40) | 46.6% (ANPL) | n/a |
| Tripathy et al., 2017 | India | National Sample Survey, 2014 | institutional delivery care | 25* (TE_Cata10) | n/a | borrowings/ sale of assets: 15% |
| **Upper middle-income countries (n=2)** | | | | | | |
| Hanna et al., 2020 | Colombia | National Health Survey, 2007 | surgical, or anaesthetic, and obstetric care | 17.4 (TE_Cata10); 10.0 (TE_Cata25) | 4.7 (ANPL) | n/a |
| Falconi & Bernabe, 2018 | Peru | National Household Survey (Encuesta Nacional de Hogares, ENAHO), 2016 | maternity care | 3.33 (CTP_Cata40) | n/a | n/a |

Note: Incidences of financial protection indicators are for the latest year of data analyzed in each study

*OOP includes direct medical and non-medical (such as transportation, food, lodging) costs; ANC = Antenatal care, PNC = postnatal care

HH = Households

CHE = Catastrophic health expenditure,

TE_CataX = CHE measured through the budget-share method; Denominator: total expenditure, Threshold: X%

TI_CataX = CHE measured through the budget-share method; Denominator: total income, Threshold: X%

nFE_CataX = CHE measured through the actual food expenditure method; Denominator: total non-food expenditure, Threshold: X%

CTP_CataX = CHE measured through the capacity-to-pay or the normative food expenditure method; Denominator: total non-subsistence expenditure, Threshold: X%

PL = Poverty line, IPL = International poverty line, ANPL = Absolute national poverty line, RNPL = Relative national poverty line,
