# Supplementary material for: Financial risk protection from out-of-pocket health spending in low- and middle-income countries: a scoping review of the literature
Source: Health Res Policy Syst. 2022 Jul 29;20:83. doi: 10.1186/s12961-022-00886-3 (PMC9336110; doi:10.1186/s12961-022-00886-3)
Supplement: Supplementary file 7 — Additional file 7. Financial risk protection through insurance and other schemes. The studies on financial risk protection through insurance and other schemes are summarized by author(s) name and year, country, data source, intervention/financial protection scheme examined, disease(s)/condition(s) (subgroup) incidences of catastrophic health expenditure, impoverishment, coping, and forgone care for financial reasons. [file 12961_2022_886_MOESM7_ESM.docx]

**Additional file 7**: Financial risk protection through insurance and other schemes

| **Study** | **Country** | **Data Source** | **Intervention/Financial Protection Scheme Examined** | **Disease(s)/ Condition(s) (subgroup)** | **Incidence of CHE (%) [CTP_Cata40, unless specified otherwise]** | **Incidence of Impoverishment (% point)** | **Incidence of Coping (%)** | **Incidence of Forgone Care for Financial Reasons (%)** |
| --- | --- | --- | --- | --- | --- | --- | --- | --- |
| **National results (n = 16)** | | | | | | | | |
| Mohanty & Dwivedi, 2021 | India | National Sample Survey (NSS), 2004 (two surveys), 2011/12, 2014, 2017/18 | no- insurance coverage vs health insurance coverage | all diseases | with insurance: 9.54%, without insurance: 9.00% | with insurance: 3.20, without insurance: 3.32 | n/a | n/a |
| Thu Thuong et al., 2021 | Vietnam | Vietnam Household Living Standards Survey, 2016 | health insurance (HI) | all diseases | with insurance: 7.37 - 15.59%, without insurance: 12.79% | n/a | n/a | n/a |
| Njagi et al., 2020b | Kenya | Household Health Expenditure and Utilisation, 2007, and 2013. | (insured vs. uninsured) | all diseases | with insurance: 4.8%, without insurance: 1.7% | n/a | n/a | n/a |
| Wang et al., 2020 | China | National Health Service Survey (NHSS), 2013 | integrated and un-integrated insurance schemes in China | all diseases | with insurance: 14.43 - 15.53%; without insurance: n/a | **at RNPL:** with insurance: 6.07 - 6.41; without insurance: n/a | n/a | n/a |
| Kazemi-Karyani et al., 2020 | Iran | Household Income and Expenditure survey (HIES), 2017 | (three years after the) Health Transformation Plan (HTP) (was launched in 2014) | all diseases | with insurance: 3.43%, without insurance: 3.04% | n/a | n/a | n/a |
| Abdi et al., 2020 | Iran | Household Income and Expenditure survey (HIES), 2014, 2015 | Health Transformation Plan (HTP) (launched in 2014) | all diseases | baseline CHE (2014): 2.9%, end line CHE (2015): 2.1 % | **at IPL UD$ 1.90/ capita/day:** baseline (2014): 0.2, end line (2015): 0.5 | n/a | n/a |
| Hernández-Vásquez et al., 2020 | Peru | National Household Survey on Living Conditions (Encuesta Nacional de Hogares, ENAHO), 2008, and 2017 |  | all diseases | with insurance: 3.6%, without insurance: 5.1% **(nFE_Cata40)** | n/a | n/a | n/a |
| Tangcharoensathien et al., 2020 | Thailand | Household Socio-Economic Survey, 1996, 1998, 2000, 2002, 2004, 2006, 2007, 2008, 2009, 2010, 2011, 2012, 2013, 2014, and 2014 | Implementation of three public health insurance schemes under the universal health insurance scheme in 2002 | all diseases | baseline (1996): 6%, endline (2015): 2% **(TE_Cata10)** | **at IPL US$ 1.9/capita/day:** baseline (1996): 1.7%, endline (2015): 0.07%; **at IPL US$ 3.1/capita/day:** baseline (1996): 1.4%, endline (2015): 0.4%; **at ANPL**: baseline (1996): 2.2%, endline (2015): 0.3% | n/a | n/a |
| Liu et al., 2019 | China | National Health Services Survey, 2013 | health insurance integration policies | all diseases | with insurance: 14.68 - 14.85%, without insurance: n/a | **at RNPL:** with insurance: 1.3 - 7.60, without insurance: n/a | n/a | n/a |
| Ahmadnezhad et al., 2019 | Iran | Household Income and Expenditure survey (HIES), 2013, 2016 | Health Transformation Plan (HTP) (launched in 2014) | all diseases | baseline (2013):2.13%, end line (2016):1.91% | n/a | no | no |
| Nikoloski & Mossialos, 2018 | Mexico | Survey of Health and Nutrition (Encuesta Nacional de Salud y Nutricion, ENSANUT), 2006, 2012 | Social Security Programs and Seguro Popular health insurance | all diseases | with insurance: approx. 2.6 - 3.2 (TE_Cata30); without insurance: 2.9 - 3.1 **(TE_Cata30)** | **at IPL US$ 1.9/capita/day**: with insurance: 0.38 -1.37, without insurance: 0.85; **at IPL US$ 3.1/capita/day:** with insurance: 1.02- 2.20; without insurance: 1.78 (IPL US$ 3.1/capita/day) | n/a | n/a |
| Falconi & Bernabe, 2018 | Peru | National Household Survey (Encuesta Nacional de Hogares, ENAHO), 2016 | health insurance | all diseases | with insurance**:** 3.65 - 4.01%; without insurance: 5.55% | n/a | n/a | n/a |
| Ahmed et al., 2018 | Vietnam | Household Living Standards Survey, 2012 | health insurance | all diseases | with insurance: 21.7 - 22.9%, without insurance: 24.2 - 28.6% | n/a | n/a | no |
| Amaya-Lara, 2016 | Colombia | Quality of Life National Survey, 2011 | insurance under the health systems | all diseases | with insurance: 8.5 - 11.8%, without insurance: 9.9% (CTP_Cata20) | n/a | n/a | n/a |
| Amponsah, 2016 | Ghana | Ghana Living Standard Survey, 1998/99, 2005/06, & 2012/13 | National Health Insurance Scheme implemented in 2005 | all diseases | baseline (1998/99): 7.62%, end line (2012/13): 5.47% **(nFE_Cata10)** | n/a | **baseline (1998/99):** saving: 7.7, borrowing from family members: 19.1, borrowing from others: 16.1; **end line (2012/13):** saving: 15.7, borrowing from family members: 2.1, borrowing from others: 8.1 | n/a |
| Narci et al., 2015 | Turkey | Household Budget Surveys, 2004 - 2010 (seven waves) | health insurance | all diseases | n/a | **at IPL: US$ 1.0/ capita/ day:** with insurance: 0.00 - 0.03; without insurance: 0.00; **at IPL: US$ 2.15/ capita/ day**: with insurance: 0.00 - 0.30; without insurance: 0.00 - 0.39; **at ANPL**: with insurance: 0.12 - 2.48; without insurance: 0.00 - 1.75; **at RNPL**: with insurance: 0.12 - 2.12; without insurance: 0.00 - 2.93 | n/a | n/a |
| **Subnational results (n = 15)** | | | | | | | | |
| Jithitikulchai et al., 2021 | Cambodia | Cambodian Socio-Economic Survey (CSEC), 2004, 2009, 2014, 2015, 2016, 2017 | Health Equity Fund (HEF) | all diseases (poor HHs) | Health Equity Fund beneficiaries: baseline (2014): 10.9%, end line (2017): 6.9% | n/a | n/a | n/a |
| Fu, 2021 | China | China Household Finance Survey (CHFS), 2015, and 2017 | Resident Basic Medical Insurance and Private Health Insurance | all disease (vulnerable HHs) | 5.65 - 15.43% | 1.14 - 5.67 | n/a | n/a |
| Wang et al., 2021 | China | China Health and Retirement Longitudinal Study (CHARLS), 2011, 2013, and 2015 | medical insurance system | all diseases (people aged 45 years and over) | with insurance: 16.3 -21.7%, without insurance: 15.5% | with insurance: 4.7 -8.4%, without insurance: 8.0% | n/a | n/a |
| Zhou et al., 2021 | China | China Health and Retirement Longitudinal Study (CHARLS), 2011, 2013, and 2015 | different insurance schemes | all diseases (people aged 45 years and over) | with insurance: 23.1 - 33.3%; without insurance: 31.2% **(nFE_Cata40)** | n/a | n/a | n/a |
| Zhang, Dong, et al., 2021 | China | China National Health Services Survey (NHSS), 2003, 2008 and 2013. | ongoing reforms in the New Cooperative Medical Scheme (NCMS) since its implementation in 2003 | all diseases, (people enrolled in NCMS) | baseline (2003): 6.7%, end line (2013): 8.7% **(TI_Cata40**) | n/a | n/a | **Individual-level analysis**: ill individual **forgone necessary (deemed by physicians) admissions** due to financial difficulties: baseline (2003): 77.5%, end line (2013): 45.4%; ill individual took **early discharge** due to financial difficulties: baseline (2003): 68.3%, end line (2013): 37.0% (2013) |
| Ma et al., 2021 | China | China Health and Retirement Longitudinal Study (CHARLS), 2015 | medical insurance system | cardiovascular diseases (people aged 45 years and over) | with insurance: 13.3 - 21.6%, without insurance: 16.3% | **at RNPL**: with insurance: 13.3 - 21.6, without insurance: 3.8 (RNPL) | n/a | n/a |
| Sun & Lyu, 2020 | China | China Family Panel Studies (CFPS), 2012, 2014, and 2016. | Urban Employee Basic Medical Insurance (UEBMI); Urban Resident Basic Medical Insurance (URBMI); New Rural Cooperative Medical Insurance (NRCMI); and Supplementary Medical Insurance (SMI) | all diseases (people aged 16 years and above) | baseline (2012): approx. 5.5 - 15.5%, end line (2016): approx. 6.0 - 16.0% **(nFE_Cata40)** | n/a | n/a | n/a |
| Ma et al., 2020 | China | China Health and Retirement Longitudinal Study (CHARLS), 2015 | medical insurance system | all diseases, (people aged 45 years and over) | with insurance: 20.5%, without insurance: 17.9% | **at RNPL**: with insurance: 7.4, without insurance: 4.8 | n/a | n/a |
| Y. Zhao et al., 2020 | China | China Health and Retirement Longitudinal Study (CHARLS), 2011, 2013, and 2015 | Health insurance schemes (UEBMI, URBMI, NRCMS, and others | NCDs (people aged 45 years and over) | with insurance: 15.1 -20.6, without insurance: 16.2% **(nFE_Cata40)** [incidence on pooled panel data from the 3 rounds] | n/a | n/a | n/a |
| Liu et al., 2017 | China | dataset for the study 'Evaluating Social Policy Supporting System for Vulnerable Families in Urban and Rural China' (ESPSS), 2014 | Medical Financial Assistance (MFA) program | all diseases (low-income households) | with medical financial assistance:62.7%, without medical financial assistance: 45.0% | n/a | n/a | n/a |
| Sun et al., 2016 | China | National Oral Health Survey, 2005 | dental insurance | dental care (individuals aged 5 - 74 years in sampled households) | with dental insurance: 0.7%, without dental insurance: 1.5% **(TE_Cata10)** | n/a | n/a | n/a |
| Pedrazzoli et al., 2021 | Ghana | National TB Patient Cost Survey, 2016 | Ghana’s National Health Insurance Scheme | TB (patients availing free treatment at public health facilities) | with insurance: 50*%, without insurance: 44*% **(TE_Cata10)** | n/a | no | no |
| Ranjan et al., 2018 | India | National Sample Survey (NSS), 2014 | publicly funded health insurance schemes | all diseases (inpatient care) | with insurance: 12.88 - 17.87%, without insurance: 18.48% (TE_Cata10) | **at ANPL:** with insurance: 6.80 - 11.66, without insurance: 13.18 | no | no |
| Goli et al., 2016 | India | National Sample Survey, 2014 | health insurance | Maternity care [women aged 15 - 49 years) | with insurance: 60.76*%, without insurance: 55.01*% | n/a | n/a | n/a |
| Doubova et al., 2015 | Mexico | Survey of Health and Nutrition (Encuesta Nacional de Salud y Nutricion, ENSANUT), 2006, 2012 | Social Security Programs and Seguro Popular health insurance | all diseases [HHs with elderly people aged 60 years and above] | with insurance: 2.02 - 3.2%, without insurance: 3.9% | n/a | savings or borrowing  or sale of assets: with insurance: 14.8 - 20.0%, without insurance: 17.9% | with insurance: 10.2 - 38.2%, without insurance: 57.8% |

Note: Incidences of financial protection indicators are for the latest year of data analyzed in each study.

NCDs = Non-communicable diseases

HH = Households

CHE = Catastrophic health expenditure,

TE_CataX = CHE measured through the budget-share method; Denominator: total expenditure, Threshold: X%

TI_CataX = CHE measured through the budget-share method; Denominator: total income, Threshold: X%

nFE_CataX = CHE measured through the actual food expenditure method; Denominator: total non-food expenditure, Threshold: X%

CTP_CataX = CHE measured through the capacity-to-pay or the normative food expenditure method; Denominator: total non-subsistence expenditure, Threshold: X%

PL = Poverty line, IPL = International poverty line, ANPL = Absolute national poverty line, RNPL = Relative national poverty line,
